# Investments for effective functionality of health systems towards Universal Health Coverage in Africa: A scoping review

**DOI:** 10.1371/journal.pgph.0001076

**Published:** 2022-09-23

**Authors:** Humphrey Cyprian Karamagi, Ali Ben Charif, Solyana Ngusbrhan Kidane, Tewelde Yohanes, David Kariuki, Maritza Titus, Charles Batungwanayo, Aminata Binetou-Wahebine Seydi, Araia Berhane, Jacinta Nzinga, David Njuguna, Hillary Kipchumba Kipruto, Edith Andrews Annan, Benson Droti

**Affiliations:** 1 Data Analytics and Knowledge Management, World Health Organization (WHO) Regional Office for Africa, Brazzaville, Republic of Congo; 2 CubecXpert, Quebec, QC, Canada; 3 Division of Policy and Planning, Ministry of Health, Asmara, Eritrea; 4 Ministry of Health, Nairobi, Kenya; 5 Independent Advisor, Windhoek, Namibia; 6 Independent Advisor, Gitega, Burundi; 7 Conmmunicable Diseases Control Division, Ministry of Health, Asmara, Eritrea; 8 Health Services Unit, KEMRI-Wellcome Trust Research Programme, Nairobi, Kenya; 9 Health Economist, Ministry of Health, Nairobi, Kenya; 10 Essential Drugs and Medicines, World Health Organization (WHO) Regional Office for Africa, Brazzaville, Republic of Congo; 11 Health Information Systems, World Health Organization (WHO) Regional Office for Africa, Brazzaville, Congo; University of Alberta, CANADA

## Abstract

The health challenges in Africa underscore the importance of effectively investing in health systems. Unfortunately, there is no information on systems investments adequate for an effective functional health system. We aimed to address this by conducting a scoping review of existing evidence following the Joanna Briggs Institute Manual for Evidence Synthesis and preregistered with the Open Science Framework (https://osf.io/bvg4z). We included any empirical research describing interventions that contributed to the functionality of health systems in Africa or any low-income or lower-middle-income regions. We searched Web of Science, MEDLINE, Embase, PsycINFO, Cochrane Library, CINAHL, and ERIC from their inception, and hand-searched other relevant sources. We summarized data using a narrative approach involving thematic syntheses and descriptive statistics. We identified 554 unique reports describing 575 interventions, of which 495 reported evidence of effectiveness. Most interventions were undertaken in Africa (80.9%), covered multiple elements of health systems (median: 3), and focused on service delivery (77.4%) and health workforce (65.6%). Effective interventions contributed to improving single (35.6%) or multiple (64.4%) capacities of health systems: access to essential services (75.6%), quality of care (70.5%), demand for essential services (38.6%), or health systems resilience (13.5%). For example, telemedicine models which covered software (technologies) and hardware (health workers) elements were used as a strategy to address issues of access to essential services. We inventoried these effective interventions for improving health systems functionality in Africa. Further analyses could deepen understanding of how such interventions differ in their incorporation of evidence for potential scale across African countries.

## Introduction

A health system “consists of all organizations, people, and actions whose primary intent is to promote, restore, or maintain health” [1]. Many health systems in countries across the world often do not meet the service requirements of their populations. Low- and middle-income countries confront the world’s most dramatic health and social services crises. In Africa, home to 54 low- and middle-income countries, health systems almost totally collapsed with the outbreak of the 2013 Ebola epidemic in West Africa for example [2]. The experience of previous disease outbreaks in Africa suggests that the health impact of coronavirus disease 2019 (COVID-19) could be challenging [3,4]. That is in addition to the sharp rise in non-communicable diseases [5,6], which is adding another burden to African health systems. Many of the fastest growing populations are observed in Africa [7], further necessitating robust systems that are fit-for-purpose, aimed to achieve greater equality, combat hunger and malnutrition, and strengthen the coverage and quality of health and education systems [8]. A quarter of the world’s population will live in Africa by 2050 [7], creating a need to push for stronger health systems, able to effectively absorb shocks created by growing epidemics of communicable and non-communicable diseases and deliver the required essential services [9].

Health systems are the foundation to ensure healthy lives and promote well-being for all at all age, the third Sustainable Development Goal established by the United Nations in 2015 [10]. However, attainment of this goal is only possible through attainment of multiple targets brought together in three interconnected themes around which results need to be attained: universal health coverage, health security, and coverage of health determinants [11]. Thus, understanding of health systems has progressed from six building blocks [1] to a focus on having complex, dynamic systems that allow for an interplay across 13 elements or investments of health systems [11]. This includes: i) three tangible hardware elements (health workforce, health products, and health infrastructure); ii) four tangible software elements (service delivery processes, health governance processes, health information systems, and financial management systems); and iii) six intangible software elements (values and norms, beliefs, practices, organizational culture, interests and networks, and relationships and power) [11]. These elements are conceptualized in the framework for health systems strengthening towards universal health coverage in the context of the third Sustainable Development Goal in Africa (also known as the Framework of Actions) [11,12]. The Framework of Actions is a reference results chain depicting the flow of action from the health system pillar areas to specific outputs, to health service outcomes, all the way to impact in the form of the achievement of the third Sustainable Development Goal. A good health system is therefore one where the interplay amongst these elements allows the operationalization of the shifts needed, to attain the expected results [11].

Strengthening a health system implies the need for improving its functionality, which can be viewed from the status of four capacities [11,13]: 1) access to essential services − capacity to overcome barriers the population may face when accessing essential services that they need; 2) quality of care − capacity to ensure the process of care provision is person-oriented and effective; 3) demand for essential services − capacity to engage with the beneficiaries, to ensure what the systems provide is aligned to their own needs, and 4) resilience of the health system − capacity to anticipate, absorb, adapt, and transform itself when facing a shock event, minimizing its impact. Due to the complex set of issues hidden behind those capacities of health systems, each capacity is deconstructed into vital signs, representing a group of sub-capacities that, taken together, constitute the overall capacities [11,13]. Use of vital signs allows a more targeted group of indicators to be selected and monitored, and also provides more granular information on where a country needs to focus within each capacity. Access to essential services includes three vital signs: physical access, financial access, and sociocultural access. Quality of care includes three vital signs: user experiences, patient safety, and effectiveness of care. Demand for essential services includes two vital signs: individual healthy actions and health-seeking behaviours. Resilience includes two vital signs: specific resilience (emergency preparedness and response capacity) and the non-specific resilience (inherent capacity of the health system). By improving in these vital signs, we assure that the delivery of essential services to the population where and when they are needed [14]. Unfortunately, health systems in the World Health Organization (WHO) African Region are performing at an average of 52.9% of what they can feasibly do. Comparing the contribution of the four capacities, all countries in the WHO African Region are underperforming, with access to essential services doing worst (46.3% of what is feasible), followed by health system resilience (48.4%), demand for essential services (51.4%), and quality of care (61.6%) [13]. Thus, there is a need for implementing, sustaining, or scaling interventions to improve the functioning of health systems for attainment of universal health coverage and the other health-related Sustainable Development Goal targets in Africa.

Unfortunately, the contribution of interventions to the effective functioning of African health systems is unclear or unknown. Thus, the WHO Regional Office for Africa sought to consolidate a menu of effective interventions that could be used to strengthen the functionality of health systems at national, sub-national, and facility (i.e., organization) levels. Here, an effective intervention refers to practices, procedures, methods, strategies, or products that have been proven effective in improving the functionality of a health system through outcome evaluations under ideal (efficacy) or real-world (effectiveness) circumstances [15].

In this knowledge synthesis, we aimed to identify existing effective interventions that contribute to strengthening the functionality of health systems at national, sub-national, or facility (organizational) levels in Africa. We hypothesized that by inventorying effective interventions that contribute to improving the four capacities of health systems, we will help to generate analysis of health systems functionality, and develop guidance for African countries on “how to” re-pivot their health system development efforts to attain expected results.

## Methods

### Design

We conducted a scoping review following the methodology recommended in the Joanna Briggs Institute (JBI) Manual for Evidence Synthesis [16]. This methodology is based on the Arksey and O’Malley framework [17] and an enhanced version developed by Levac and colleagues [18]. Scoping reviews are defined as “a type of evidence synthesis that aims to systematically identify and map the breadth of evidence available on a particular topic, field, concept, or issue, often irrespective of source (i.e., primary research, reviews, non-empirical evidence) within or across particular contexts” [19]. We used an online tool to identify relevant resources for designing this review [20]. We pre-registered the protocol of this review with the Open Science Framework (OSF) on May 19, 2022 (registration identifier: https://osf.io/bvg4z). We reported this review according to the Preferred Reporting Items for Systematic Reviews and Meta-Analysis (PRISMA) extension for Scoping Reviews (PRISMA-ScR) checklist (**S1 Appendix**) [21]. In this manuscript, the noun “report” refers to a document (paper or electronic) supplying information about a study and the noun “record” refers to the title or abstract of a report indexed in a database or website [22].

*Integrated knowledge translation approach* [23]: our review involved extensive participation of female and male international experts as equal members of the core team. Team members represented key stakeholder groups across African countries (e.g., Republic of Congo, Comoros, Eritrea, Kenya, Burundi, Namibia, Uganda, and South Africa), including decision makers, clinicians, and researchers. No patient or member of the public was involved in this work. Team members met weekly to discuss the progress of work during the current week. We used a virtual teamwork space using Google Drive and communicated using Microsoft Teams and WhatsApp. We also conducted three multidisciplinary consultations with knowledge users from the WHO Regional Office for Africa to receive theoretical, conceptual, or practical insights for guiding the interpretation and dissemination of findings.

### Eligibility criteria

Following the “PICOS” (participants, intervention, comparator, outcome, and setting) framework [24], we used the following inclusion criteria:

- **Participants (P):** We included any organization (e.g., geographical regions, clinical sites, communities) or system (e.g., district health systems) involved in the delivery or receipt of health care or services.
- **Intervention (I):** We included any interventions that covered at least one of the 13 elements of health systems. We included research describing the development of an intervention, the evaluation of the efficacy, efficiency or effectiveness of an intervention, or the implementation of an intervention [25]. We included those interventions regardless of its maturity stage [25], but we drew a distinction between an effective and non effective intervention [15]. In the context of this review, an effective intervention refers to any practice, procedure, method, strategy, or product that has been reported effective by the authors of the included study in improving a capacity of a health system.
- **Comparator (C):** We considered both studies with comparison groups and studies without (i.e., no restriction).
- **Outcomes (O)**: We considered any metrics or indicators related to at least one of the four capacities for a functional health system: 1) access to essential services, 2) quality of care, 3) demand for essential services, and 4) resilience of the health system [11].
- **Setting (S)**: We considered any country in the African continent and any low-income or lower-middle-income countries in other regions according to the 2022 World Bank classifications [26]. Health systems in high-income countries (e.g., Canada) and upper-middle-income countries outside Africa (e.g., Argentina) have their own organizational cultures, policies, and traditions, exacerbating challenges of transferability, implementation, sustainability, and scaling of their interventions in Africa. This distinction is important because effectiveness in those countries may not necessary be achieved in Africa.

In other words, we included any empirical research describing an intervention that contributed to improving any capacity of health systems in Africa or in any other low-income or lower-middle-income regions outside Africa (**Table 1**).

**Table 1:** Criteria for considering records or reports for this review.

Criteria	Inclusion	Exclusion
Type of record or report	◻ Any empirical research: ◻ Original study ◻ Evaluation study ◻ Knowledge synthesis ◻ Government document	◻ Editorial (commentary, letter, note) ◻ Conference abstract ◻ Protocol ◻ Retraction ◻ Non-English and non-French reports
Participants	◻ Any organization (e.g., regions, clinical sites, communities) or system (e.g., district health system) involved in the delivery or receipt of health care or services	◻ The intervention is not intended to be used for a health organization or a health system ◻ The intervention is not intended to be used in the field of health
Intervention	◻ Any intervention (e.g., practice, product, procedure, strategy, method) covering at least one of the 13 elements of health systems	◻ The study did not describe an intervention ◻ The intervention did not cover any of the 13 elements of health systems
Outcomes	◻ Any metrics or indicators related to at least one of the four capacities of health systems: ◻ Access to essential services ◻ Quality of care ◻ Demand for essential services ◻ Resilience of a health system	◻ The intervention is not intended to be used to improve capacities of health systems in terms of access to essential services, quality of care, demand for essential services, or resilience of a health system
Setting	◻ Any African country ◻ Any low-income region ◻ Any lower-middle-middle region	◻ Only high-income or upper-middle-income regions outside Africa were targeted for the intervention

### Literature search

We performed a comprehensive search to identify records through both electronic databases of peer-reviewed literature and secondary searches using other relevant sources. No restrictions regarding date of publication, language, place of publication, or type of reports were applied to our search strategy.

First, we searched the following seven electronic databases from their dates of inception to the final search date of May 3, 2022 (MEDLINE via Ovid, PsycINFO via Ovid, Cochrane Library, CINAHL via EBSCOhost, and ERIC via Ovid) or May 9, 2022 (Web of Science and Embase via Elsevier). ABC drafted the preliminary version of the search strategy for Ovid MEDLINE. The search terms were based on previous works to reflect three concepts: 1) intervention [27,28], 2) health system functionality [11,29], and 3) low- and middle-income regions [30,31]. For the latter, we used a search filter developed by the Cochrane Effective Practice and Organisation of Care (EPOC) in collaboration with the WHO and Campbell Collaboration [30,31]. The preliminary search strategy was reviewed by our core team of experts in health information systems, public health, implementation science, or knowledge syntheses from Africa (TY, DK, MT, CB, SNK, and MMR). The search terms were adapted to the above-mentioned databases. Details of the search strategy in each electronic database can be found in can be found in **S2 Appendix**.

Second, we identified other relevant reports by searching the WHO Library, Google Scholar, Google, and Hinari in English or French. We used multiple combinations of search terms related to the concepts of intervention, health system functionality, and low- and middle-income regions (**S3 Appendix**). We screened at least the first 30 results for each search, a threshold often used to analyse medical content available on websites. Indeed, results lower down the relevancy lists are often duplications of earlier results and it is rare for users to click past the third page of ten search results per page [32,33].

### Study selection process

We operationalized our inclusion criteria based on our PICOS elements. After removal of duplicates, ABC performed a calibration exercise and discussed with team members (SNK, TY, DK, MT, and CB) to ensure the criteria captured relevant studies. One reviewer (ABC, TY, DK, MT, CB, AB, JN, or DN) screened records and reports for relevance and selected eligible reports using the eligibility criteria. Each record or report was screened by one of those reviewers and checked by another (ABC, TY, or AB). All disagreements were resolved through discussion between the reviewer and the checker. For each ineligible report, we documented a reason for the exclusion. Records that referred to the same report were considered duplicates, but records that referred to reports that were merely similar were considered unique [22]. We used EndNote 20 software to identify and removed duplicates as well as standardized forms in Google Sheets for the selection process.

### Data collection process

We developed a form in Google Sheets to guide extraction of variables based on the Framework of Actions [12]. ABC performed a calibration exercise and discussed with team members (SNK, TY, DK, MT, and CB) to ensure the form captures relevant data. Eight reviewers (ABC, TY, DK, MT, CB, AB, JN, and DN) independently extracted data using this standardized form. Each included report was independently extracted by two of those reviewers. All disagreements were resolved through discussion with a third party (HCK, ABC, SNK, TY, AB, JN, or DN). Information extracted from each included report was:

■ Study characteristics (e.g., title, authors, and year of publication)
■ Characteristics of interventions (e.g., name, objective, scope of actions, level of the intervention scope, elements of health systems covered by the intervention, capacities of health systems targeted by the intervention, setting of the interventions). Each intervention was mapped to the varying level of contribution to one capacity over another using four-point Likert scale: “none,” “minimal,” “somewhat,” and “mainly.”

### Risk of bias

Due to the nature of our research question, we did not perform an appraisal for risk of bias. This is consistent with the Joanna Briggs Institute (JBI) Manual for Evidence Synthesis [16].

### Data analysis

We summarized data using a narrative approach involving framework and content analysis. A unique report could include more than one intervention, but the individual intervention was the unit of analysis. We used framework analysis to classify data according to pre-defined categories and content analysis to organize qualitative data by counting and reporting the frequency of categories found. We analysed both elements and capacities of health systems through a meticulous examination of qualitative data to identify patterns, themes, or inferences. We used the PRISMA 2020 flowchart to describe the process of report selection [22]. We summarized the main characteristics of interventions, including elements of health systems covered by the intervention, capacities of health systems targeted by the intervention, and levels of intervention scope (national, sub-national, or facility levels) in a tabular display using SAS 9.4 software [34].

## Results

### Selection of interventions

**Fig 1:**
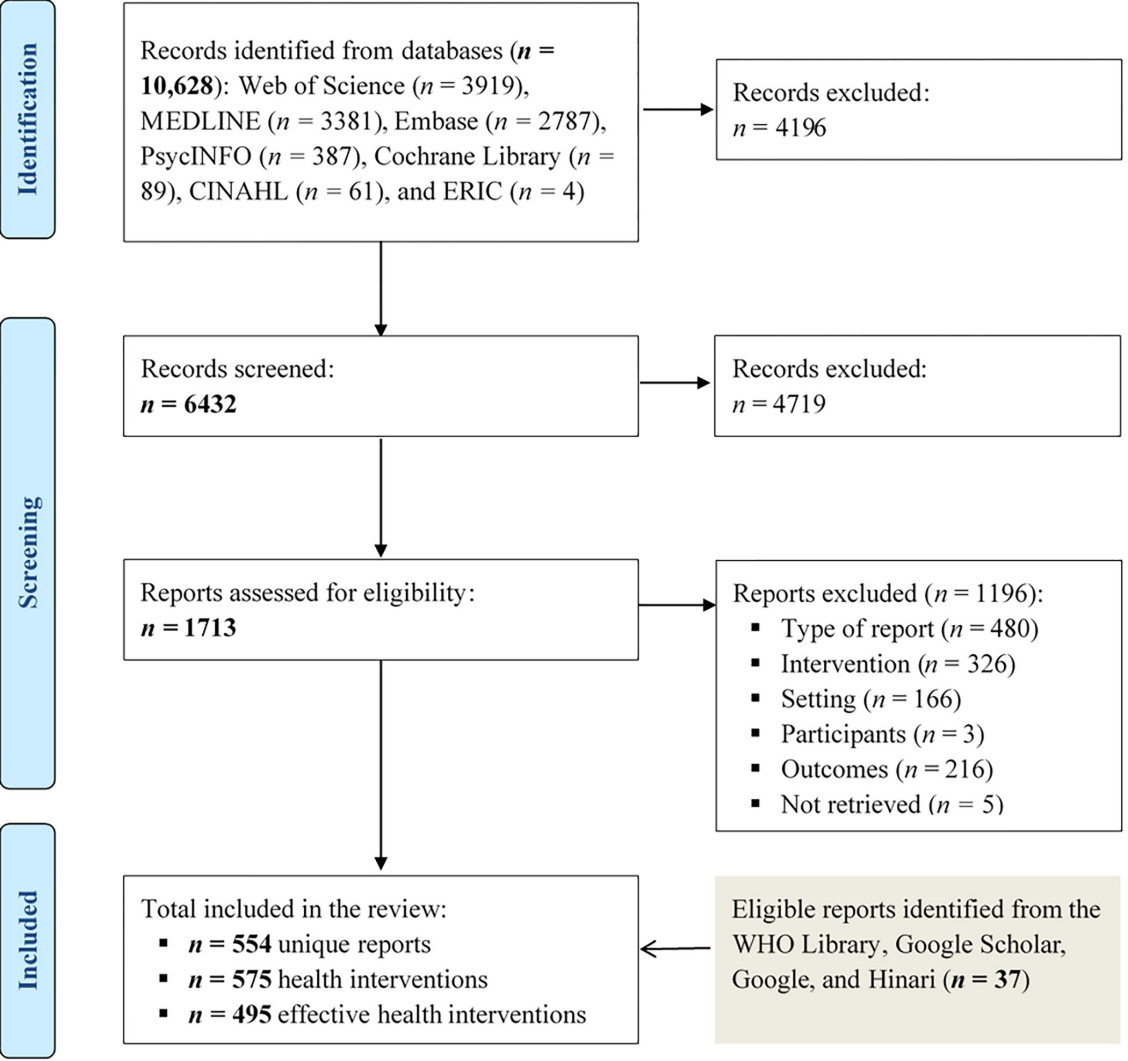
PRISMA 2020 flow diagram of the intervention inclusion process.

Our electronic search identified 10,628 potentially relevant records. Of these, 4196 were duplicates, leaving 8494 records. Of these, 4719 did not meet the review criteria. Thus, we reviewed a total of 1713 reports, retained 517, and excluded 1196. Reasons for exclusion included: wrong type of report, no eligible intervention, no health organization or system targeted by the intervention, no metric related to a capacity of a health system, and wrong setting (**S4 Appendix**). In addition, our secondary searches led to the inclusion of 37 additional reports. Overall, we included a total of 554 unique reports from all sources, which described a total of 575 interventions or packages of interventions, of which 495 were reported effective in improving the functionality of health systems (**Fig 1**). Descriptions of each intervention can be found in the appendix (**S5 Appendix**).

### Characteristics of included interventions

Main characteristics of included interventions are outlined in **Table 2**. Most interventions were developed or implemented in Africa (*n =* 465, 80.9%), especially in South Africa (*n =* 52, 11.2%), followed by Ethiopia (*n =* 47, 10.1%), Uganda (*n =* 47, 10.1%), Tanzania (*n =* 45, 9.7%), Kenya (*n =* 40, 8.6%), and Nigeria (*n =* 38, 8.2%) (S5 Appendix). Most interventions were developed or implemented on a sub-national level (*n =* 317, 55.1%), followed by national (*n =* 287, 49.9%) and facility (*n =* 266, 46.3%) levels.

**Table 2:** Characteristics of included interventions.

Characteristic			Total (*n =* 575)	Levels of intervention scope		
				National (*n =* 287)^2^	Sub-national (*n =* 317)^2^	Facility (*n =* 266)^2^
**Region** ^1^			***n* (% column)**	***n* (% column)**	**n (% column)**	***n* (% column)**
	Africa		371 (64.5)	175 (61.0)	235 (74.1)	196 (73.7)
	Outside Africa		110 (19.1)	47 (16.4)	58 (18.3)	38 (14.3)
	Africa and outside		94 (16.3)	65 (22.6)	24 (7.6)	32 (12.0)
**Evidence of efficacy or effectiveness** ^1^			***n* (% column)**	***n* (% column)**	**n (% column)**	**n (% column)**
	Established		495 (86.1)	246 (85.7)	273 (86.1)	238 (89.5)
	Unknown		80 (13.9)	41 (14.3)	44 (13.9)	28 (10.5)
**Health system element covered** ^2^			***n* (% row)** ^2^	***n* (% row)** ^2^	***n* (% row)** ^2^	***n* (% row)** ^2^
	** Tangible software**		512 (89.0)	259 (90.2)	285 (89.9)	251 (94.4)
		Service delivery processes	445 (77.4)	225 (78.4)	245 (77.3)	226 (85.0)
		Health governance processes	214 (37.2)	126 (43.9)	113 (35.6)	115 (43.2)
		Health information systems	208 (36.2)	108 (37.6)	124 (39.1)	129 (48.5)
		Financial management systems	107 (18.6)	61 (21.3)	61 (19.2)	60 (22.6)
	** Tangible hardware **		450 (78.3)	229 (79.8)	253 (79.8)	230 (86.5)
		Health workforce	377 (65.6)	192 (66.9)	222 (70.0)	189 (71.1)
		Health infrastructure	175 (30.4)	82 (28.6)	96 (30.3)	104 (39.1)
		Health products	173 (30.1)	84 (29.3)	96 (30.3)	112 (42.1)
	** Intangible software**		269 (46.8)	130 (45.3)	153 (48.3)	134 (50.4)
		Practices	202 (35.1)	94 (32.8)	117 (36.9)	113 (42.5)
		Values and norms	113 (19.7)	55 (19.2)	69 (21.8)	74 (27.8)
		Relationships and power	104 (18.1)	59 (20.6)	54 (17.0)	49 (18.4)
		Organizational culture	100 (17.4)	49 (17.1)	55 (17.4)	64 (24.1)
		Interests and networks	84 (14.6)	48 (16.7)	48 (15.1)	44 (16.5)
		Beliefs	84 (14.6)	35 (12.2)	55 (17.4)	64 (24.1)
	**Number of elements covered**					
		Median ± interquartile range	3.0 ± 4.0	3.0 ± 4.0	3.0 ± 4.0	3.0 ± 4.0

**Notes:**^**1**^Percentages in the column may add up to ± 100% because of rounding error.

^**2**^The numbers or percentages in the row or in the column do not add up since they are not mutually exclusive.

### Elements of health systems covered by the included interventions

Most interventions covered three or more elements of health systems (median: 3 elements, interquartile range: 4): tangible software (*n =* 512, 89.0%), tangible hardware (*n =* 450, 78.3%), or intangible software (*n =* 269, 46.8%) (Table 2). For example, telemedicine models covered tangible software (e.g., technologies) and tangible hardware (e.g., health workers) elements and were used as a strategy to address issues of access and availability of speciality care in India [35].

For those that covered tangible software elements, 445 (77.4%) were related to service delivery processes, 214 (37.2%) were related to health governance processes, 208 (36.2%) were related to health information systems, and 107 (18.6%) were related to financial management systems. Predominantly, these interventions focused on identifying, understanding, or overcoming barriers that affect the effectiveness of health service delivery with emphasis on maternal and child health, communicable diseases, and the emergence of noncommunicable diseases (e.g., [36–39]) (S5 Appendix).

For those that covered tangible hardware elements, 377 (65.6%) were related to the health workforce, 175 (30.4%) were related to health infrastructure, and 173 (30.1%) were related to health products. Predominantly, these interventions focused on overcoming barriers the population may face when accessing essential services that they need. This included increasing the numbers and skills of the available human resources for improving the capacity of the health system to respond to population needs. For example, community health workers were trained in multiple countries through the Structured Operational Research and Training IniTiative (SORT IT), building operational research capacity for improving public health and skills to mitigate the health system effects of the COVID-19 pandemic [40] (S5 Appendix).

With regards to the intangible software elements, 202 (35.1%) were related to practices, 113 (19.7%) were related to values and norms, 104 (18.1%) were related to relationships and power, 100 (17.4%) were related to organization culture, 84 (14.6%) were related to interests and networks, and 84 (14.6%) were related to beliefs. Predominantly, those interventions focused on: 1) preparedness, response, coverage, and programs (e.g., [41,42]); 2) quality improvement, benchmarking, and accreditation (e.g., [43–46]); 3) health worker management and supervision including availability and distribution, workload, capacity, salaries, benefits, and motivation (e.g., [47–50]); 4) evidence-based decision-making to enhance governance and policy-making for resilience including the use of health informatics, mobile health, and performance-based financing (e.g., [51–54]); 5) behaviour change communication (e.g., [55,56]); and 6) programs and reforms such as family medicine, sub-national health systems, task-shifting, and capacity building (e.g., [57–59]) (S5 Appendix).

### Contribution of effective interventions to the functionality of health systems

There were 495 interventions (86.1%) for which evidence of efficacy or effectiveness for functionality of health systems has been established (Table 1). Those effective interventions contributed to improving either single (*n =* 176, 35.6%) or multiple (*n =* 319, 64.4%) capacities of health systems, including access to essential services (*n =* 374, 75.6%), quality of care (*n =* 349, 70.5%), demand for essential services (*n =* 191, 38.6%), and resilience of health systems (*n =* 67, 13.5%) (**Table 3**). Effective interventions that contributed to improving each capacity were mostly related to health service delivery (*n =* 401, 81.0%) and health workforce (*n =* 329, 66.5%). Through the examination of qualitative data, we found that effective interventions commonly focused on the following cross-cutting themes: mobilization of health workers, community involvement, capacity building, digital health systems, and financial systems (S5 Appendix).

**Table 3:** Characteristics of effective interventions.

		Effective interventions that contributed to improving:								
		All^2^	Access to services		Quality of care		Demand for services		Resilience to shocks	
			Mainly^3^	All^2^	Mainly^3^	All^2^	Mainly^3^	All^2^	Mainly^3^	All^2^
Effective interventions		*n =* 495	*n =* 193	*n =* 374	*n =* 193	*n =* 349	*n =* 39	*n =* 191	*n =* 30	*n =* 67
**Element of health systems**		***n* (% column**)^1^								
**Tangible software**		451 (91.1)	185 (95.9)	343 (91.7)	18 (93.3)	327 (93.7)	37 (94.9)	181 (94.8)	27 (90.0)	62 (92.5)
	Service delivery processes	401 (81.0)	166 (86.0)	310 (82.9)	169 (87.6)	303 (86.8)	31 (79.5)	167 (87.4)	24 (80.0)	51 (76.1)
	Health governance processes	188 (38.0)	76 (39.4)	156 (41.7)	76 (39.4)	144 (41.3)	16 (41.0)	97 (50.8)	15 (50.0)	39 (58.2)
	Health information systems	185 (37.4)	70 (36.3)	134 (35.8)	81 (42.0)	136 (39.0)	16 (41.0)	81 (42.4)	17 (56.7)	34 (50.7)
	Financial management systems	95 (19.2)	45 (23.3)	81 (21.7)	35 (18.1)	70 (20.1)	11 (28.2)	55 (28.8)	10 (33.3)	20 (29.9)
**Tangible hardware **		391 (79.0)	163 (84.5)	305 (81.6)	161 (83.4)	282 (80.8)	28 (71.8)	155 (81.2)	26 (86.7)	53 (79.1)
	Health workforce	329 (66.5)	130 (67.4)	253 (67.6)	144 (74.6)	243 (69.6)	24 (61.5)	133 (69.6)	22 (73.3)	45 (67.2)
	Health products	160 (32.3)	72 (37.3)	126 (33.7)	73 (37.8)	130 (37.2)	12 (30.8)	83 (43.5)	12 (40.0)	26 (38.8)
	Health infrastructure	156 (31.5)	68 (35.2)	126 (33.7)	59 (30.6)	117 (33.5)	12 (30.8)	71 (37.2)	15 (50.0)	29 (43.3)
**Intangible software**		241 (48.7)	19 (47.2)	185 (49.5)	97 (50.3)	177 (50.7)	24 (61.5)	134 (70.2)	19 (63.3)	43 (64.2)
	Practices	184 (37.2)	70 (36.3)	145 (38.8)	71 (36.8)	136 (39.0)	18 (46.2)	105 (55.0)	17 (56.7)	34 (50.7)
	Values and norms	101 (20.4)	36 (18.7)	80 (21.4)	43 (22.3)	79 (22.6)	13 (33.3)	72 (37.7)	7 (23.3)	16 (23.9)
	Relationships and power	91 (18.4)	33 (17.1)	68 (18.2)	34 (17.6)	61 (17.5)	13 (33.3)	51 (26.7)	9 (30.0)	20 (29.9)
	Organizational culture	90 (18.2)	28 (14.5)	66 (17.6)	45 (23.3)	73 (20.9)	8 (20.5)	57 (29.8)	7 (23.3	19 (28.4)
	Beliefs	80 (16.2)	30 (15.5)	64 (17.1)	32 (16.6)	58 (16.6)	14 (35.9)	64 (33.5)	5 (16.7)	11 (16.4)
	Interests and networks	75 (15.2)	26 (13.5)	56 (15.0)	30 (15.5)	53 (15.2)	12 (30.8)	44 (23.0)	6 (20.0)	15 (22.4)
**Number of elements**		**Median ± interquartile range**								
	Score ranging from 1 to 13	4.0 ± 4.0	4.0 ± 4.0	4.0 ± 4.0	4.0 ± 3.0	4.0 ± 4.0	5.0 ± 4.0	5.0 ± 5.0	5.5 ± 5.0	5.0 ± 5.0
**Number of capacities**		***n* (% column**)^1^								
	1	176 (35.6)	52 (26.9)	76 (20.3)	60 (31.1)	78 (22.3)	6 (35.9)	7 (3.7)	11 (36.7)	15 (22.4)
	2	174 (35.2)	72 (37.3)	153 (40.9)	74 (38.3)	128 (36.7)	14 (35.9)	50 (26.2)	8 (26.7)	17 (25.4)
	3	123 (24.8)	64 (33.2)	123 (32.9)	49 (25.4)	121 (34.7)	18 (46.2)	**112 (58.6)**	7 (23.3)	13 (19.4)
	4	22 (4.4)	5 (2.6)	22 (5.9)	10 (5.2)	22 (6.3)	1 (2.6)	22 (11.5)	4 (13.3)	22 (32.8)
	Median ± interquartile range	2.0 **±** 2.0	2.0 **±** 2.0	2.0 **±** 1.0	2.0 **±** 2.0	2.0 **±** 1.0	2.0 ± 1.0	3.0 **±** 1.0	2.0 **±** 2.0	3.0 ± 2.0

**Notes:** ^1^The numbers or percentages in the row or in the column do not add up since they are not mutually exclusive.

^2^Effective intervention that *mainly* contributed to improve the capacity.

^3^All effective interventions that contributed to improve the capacity (mainly, somewhat, minimal contribution).

### Contribution of effective interventions to each capacity of health systems

**Fig 2:**
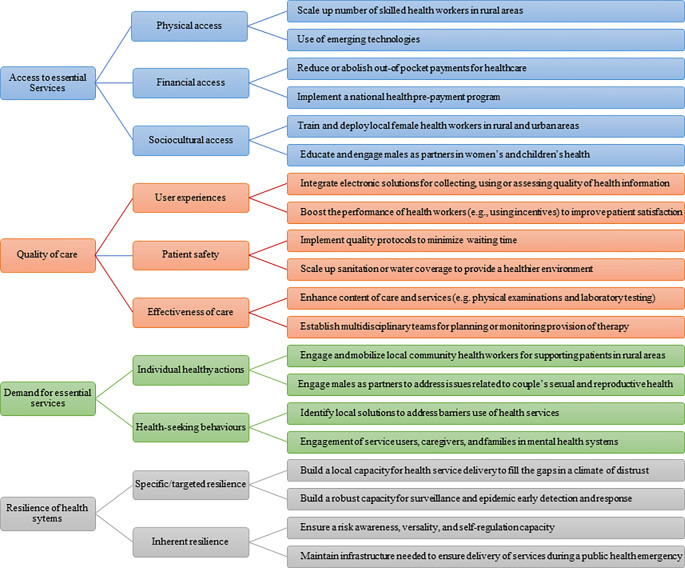
Examples of effective interventions that contributed to improving the functionality of health systems.

**Fig 2** provides examples of effective interventions that contributed to improving capacities of health systems in Africa or other low-income or lower-middle-income regions.

### Improving access to essential services

For effective access to essential services, interventions mainly focused on integration of community health workers into the primary healthcare system, digital health systems, financial systems based on vouchers, vehicle support, and involvement of high-level leadership. For example:

■ *To improve physical access*, countries scaled a number of skilled community health workers for routine and emergency services in rural areas [60–62]. Also, health technologies were used to improve healthcare service delivery and overarching goal of universal health coverage [35,63,64].
■ *To improve financial access*, countries implemented financing mechanisms (e.g., prepayment and risk pooling) to reduce or abolish out-of-pocket payments for healthcare, especially fees charged at the point of use for essential healthcare [65,66]. The Sierra Leone Free Health Care Initiative (FHCI) was a way to remove barriers to financial access and to improve coverage and equity of essential services for mothers and children [65].
■ *To improve sociocultural access*, countries trained and deployed female health workers recruited from their prospective villages to limit the high staff turnover and address gender, social and cultural factors, and thus provide services acceptable to each community [49]. Also, countries educated and engaged males as partners in women’s and children’s health to address issues such as gender-based violence, early marriage, and the reproductive health needs of adolescents [67].

### Improving quality of care

For effective quality of care, interventions mainly focused on improving training of health workers, user involvement, accreditation approaches, case detection and referrals through community and outreach services, ambulance drivers on first aid, management and oversight at district level, equipping health facilities with basic emergency obstetric care gadgets, introduction of clinical guidelines and protocols for diagnosing and managing most common obstetric emergencies, monitoring systems to identify antenatal complications, reminding for antenatal care attendance, and mobile health applications. For example:

■ *To improve user experiences*, countries implemented integrated electronic mechanisms on a larger scale for collecting, using, and assessing quality of routine health information [68]. Also, countries supported community and facility evidence-based and district-wide health systems strengthening interventions to reduce maternal and perinatal mortality by improving quality of care [69,70].
■ *To improve patient safety*, countries implemented processes or quality appointment scheduling protocols that keep appointment waiting times at a minimum [71]. When patients cannot access necessary treatment, they may become increasingly sick while waiting for an appointment. In the same way, countries scaled sanitation or water coverage to provide a healthier environment to children and save lives of children under five (e.g., reduce diarrheal incidence or incidence of acute respiratory infections) [72].
■ *To improve effectiveness of care*, countries improved the content of care provided (e.g. physical examinations, laboratory testing and waiting time) through supplying medical consumables, equipment and laboratory reagents or facilities, seminars and on-job training for health professionals, adoption of WHO guidelines, development health education materials, workshops with patients and supervision of providers [73,74]. Also countries established multidisciplinary teams for planning or monitoring provision of essential service packages to accelerate highly active antiretroviral treatment coverage [75].

### Improving demand for essential services

For effective demand for essential services, interventions mainly focused on mobilization of health worker models, capacity building, performance-based financing programs, and leadership and governance practices. For example:

■ *To improve individual healthy actions*, countries ensured involvement of community health workers to support services to patients with communicable or non-communicable disease in rural areas [76,77]. Countries also educated and engaged males as partners in women’s and children’s health to address issues related to couple’s sexual and reproductive health [67].
■ *To improve health-seeking behaviours*, countries identified and implemented local measures to address barriers to the effective implementation of immunization programs both on the supply and demand side (e.g., low awareness level among health workers regarding vaccine-preventable diseases and their risks, belief in and use of local remedies for prevention and treatment) [78]. Also, countries implemented evidence-based models and processes for meaningfully and equitably involving service users, caregivers or families in mental health systems [76,79].

### Improving resilience of health systems

Resilience to external shocks has rarely been the domain of focus, except in the cases of Ebola and the pandemic of coronavirus disease 2019 (COVID-19). Beyond service delivery processes and health work force mobilization, practices (*n =* 17, 56.7%), health information systems (n = 17, 56.7%), health infrastructure (*n =* 15, 50%), and health governance processes (n = 15, 50.0%) were covered largely by effective interventions that mainly contributed to resilience of health systems (Table 3). For effective resilience of health systems, interventions mainly focused on government support, system preparedness, response and service coverage programs, health worker availability and distribution, and evidence-based decision making to enhance governance and policy making for resilience including the use of health informatics, mobile health, and performance-based financing. For example:

■ *To improve specific or targeted resilience*, countries updated their curricula of the community health workforce to align training to needs and build operational research capacity for strengthening health care delivery systems, improving program performance and promoting public health [80–82]. Also, countries developed an annual report on the state of surveillance in line with recommendations of the international health regulations to demonstrate that enhancements could be made in a short period to the capacity for surveillance and early detection of and response to disease outbreaks. Achievements could include enhanced laboratory testing capability for several priorities, established emergency operations functions, demonstration of the need and capability for information systems to enhance public health emergency reporting [55].
■ *To improve non-specific resilience*, countries strengthened community health programs through the scaling of community health and the removal of bottlenecks such as: a) poor governance and weak management, b) frequent stock-out of medicines, c) poor data management, d) ineffective supervision, e) lack of motivation or incentives, f) limited community involvement and g) fragmented programming [82]. Also, countries maintained critical infrastructure (e.g., water, sanitation, transport) [83] and an adequate health workforce (e.g., training, support) [84] needed to ensure continued functioning during a public health emergency.

## Discussion

We identified existing interventions that could contribute to capacities of health systems in Africa and other low-income or lower-middle-income regions. Most interventions were developed or implemented in Africa, covered multiple elements of health systems, and focused on service delivery at sub-national levels. The interventions that showed efficacy or effectiveness in improving the four capacities were mostly related to service delivery processes and health workforce mobilization. These findings lead us to make the following observations.

*First*, most included interventions covered multiple elements with a focus on identifying, understanding, or overcoming service delivery barriers at sub-national level. This result suggests that service delivery touch as many as elements of the health system to bring substantial improvements in strengthening capacities of health systems at sub-national level. In fact, inefficient service delivery results from a combination of multiple causes including inadequate health products, inefficient human resources, corruption, and under-utilization of infrastructure [85]; whereas adequate service delivery results from a successful combination of the other elements of health systems. Generally, the 13 elements of health systems should not be seen as silos because they are integral parts of the same puzzle, and if one is left out, the whole arrangement may not function properly. For example, health financing and its management is crucial to ensure availability of health products to be delivered, purchase necessary equipment, and fund payments to staff [84]. The key considerations to health service provision in low- and middle-income countries are often linked to resource management and the use of health infrastructure such as technologies, electricity, and voucher schemes. Service delivery is considered good when equitable access to a comprehensive range of high-quality health services is ensured within an integrated and person-oriented continuum of care [86]. Health systems are only as effective as the services they provide [87]. As all other elements support the delivery of health services, disruption in these other elements will indirectly impact on the quality of delivery. Thus, there is a need for multi-pronged interventions involving tangible and intangible elements for improving each capacity of health systems. For example, we found that service delivery processes, health workforce mobilization, practice changes, health information systems, health infrastructure, and modes of governance were all elements capable of producing both intended and unintended consequences for health systems resilience. Thus, the cross-cutting nature and dynamic relationship between service delivery and the other elements of health systems created challenges in isolating a particular intervention to describe overall implications and recommendations for each element. The development of validated definitions and filters for these elements would be of value for future works in the field.

*Second*, the effective interventions related to service delivery processes and health workforce mobilization contributed to improving the four capacities of health systems. This suggests that service delivery and health workforce were the critical tangible elements of health systems in improving the functionality of health systems in low- and middle-income countries. Given the centrality of the health workers for delivering high-quality of essential services (quality and access) that respond to community needs (demand) when facing a shock event or not (resilience) in underserved areas, service delivery and health workforce will remain critical elements in improving overall health systems performance in low- and middle-income countries. The four capacities of health systems could mainly differ in accordance with elements related to health service delivery (e.g., delayed test results or appointment, difficulty navigating referral system, long wait for transfer or treatment, geographical inaccessibility, long travel distance, and lack of primary care service) or health workforce (e.g., poor attitude, mismanagement, misdiagnosis, lack of knowledge, strike, and unavailability of doctors) [88]. A competent health workforce is a vital resource for health services delivery and health systems can only function with health workers; the improvement of the four capacities of health systems is therefore dependent on their availability, accessibility, acceptability, and quality. We found that most effective interventions were undertaken to increase health workers numbers, performance, knowledge, and skills in order to address to population needs and reduce disparities across urban and rural areas [40,50,63,67,81,89,90]. Community health workers may be faced with inadequate access to training and reference materials, poor-quality communication systems for feedback from experts or supervisors in the diagnosis and management of complex cases, and difficulty maintaining patients within the continuum of care through follow-up visits or referrals, thereby impacting the access to high-quality of essential services they can deliver. There is need to align their curricula and build operational research capacity for strengthening service delivery systems and improving health system performance. For example, for effective resilience of health systems, some countries engaged, trained, and mobilized both female and male local health workers who are trusted by the community and understand the social and cultural complexities of the population, to fill the gap created by conventional health workers in a climate of distrust, where the latter were reluctant to treat patients, sick people were afraid to self-identify and caregivers were afraid to take children to the clinic [40,81]. Notably, we found that digital health has been leveraged to mitigate some of these challenges community health workers may face in low- and middle-income countries. An initiative of mobile consulting in Bangladesh, Kenya, Nigeria, Pakistan, and Tanzania, was explored to assess whether it is a viable option for communities with minimal resources, showing that there are indications of local readiness for mobile consulting in communities with minimal access to essential services, and that mobile consulting had the potential to strengthen health systems during and beyond the COVID-19 global pandemic [89]. The introduction of Millennium Villages Global Network (MVG-Net) in multiple African countries such as Ghana, Rwanda, Tanzania, and Uganda has facilitated point-of-care decision support through mobile phone systems based on a short message service [90]. This program assisted community health workers in real-time monitoring of their pre-determined community-based services, hence enhancing quality service delivery at community level. The Mobile Midwife, a mobile application implemented in Ghana in 2010, sends timely messages in local languages to register expectant mothers and new parents [63]. Thus, the use of mobile phone systems in low- and middle-income countries has taken place faster than any other infrastructural development. However, the field of digital health is severely under-researched in Africa, yet it can be an alternative for strengthening health systems and the ways in which health services are delivered. There is growing evidence that strengthening health workforce through digital health strategies can contribute to ensuring that populations have access to high quality, responsive, and sustainable health system for otherwise under-served populations in low- and middle-income countries [86,91,92]. African health systems are underperforming in all capacities, and countries need to prioritize on effective interventions (e.g., digital health models and training programs) related to health workforce mobilization and service delivery processes, which are critical tangible elements for improving functionality of health systems in low- and middle-income countries. The WHO Regional Office for Africa has proposed the adoption of a digital health platform to streamline different solutions to a cohesive whole [93].

*Third*, there is widespread enthusiasm for scaling effective interventions to improve functionality of African health systems [11,27], but we know little about the scalability of identified interventions. By scalability, we mean the ability of an effective intervention to change in size, while retaining effectiveness [94,95]. Pitfalls, problems, and difficulties with scaling even proven interventions suggest that it might be beneficial to identify phases as well as components of scaling of our included interventions [96]. Indeed, a previous knowledge synthesis found that 40% of scaled public health interventions had not been trialled in real world [97]. Other studies showed that coverage of effective interventions (i.e., extent to which people, organizations, or systems adopt the intervention with fidelity) was least likely to be assessed [28,98]. Yet, for interventions to have a substantial impact, they need to be adopted by a large enough population over a sustained period and one of the potential drawbacks is that some of our included effective interventions might have ceased to exist since they might have been project or donor based. Also, interventions often need considerable adaptation to enable implementation at scale, a process that can reduce or remove the effects of interventions [99]. Assessing the scalability of identified interventions is important to determine whether significant investments in their scaling will achieve worthwhile benefits to the community. Thus, there is a need for a tool to help rate and rank our included interventions for their scalability across African countries. A systematic review published a useful inventory of tools, components, and items for assessing the scalability of interventions in health as well as interpretability criteria that could be used to weed out poor items [27]. This will help to propose a scalability assessment tool aligned with the Framework of Actions and to select effective interventions that could be successfully utilized to improve the functionality of health systems in African countries.

*Finally*, we acknowledged that caution should be exercised when interpreting the implications of our findings. First, the criteria for what constitutes an evidence-based intervention are not met by some interventions listed in our inventory [15]. However, at this early stage of this inventory, our interest is in creating an intervention pool and mapping the available evidence for strengthening functionality of health systems in Africa. Thus, we aimed to be as inclusive as possible as nothing can be done after the fact to compensate for interventions we neglected to include. Second, in the same implementation project, several interventions may be employed across the life of the project and these interventions could cover multiple elements of health systems. Thus, it may be difficult to isolate a particular intervention as it may relate to tangible and intangible elements. However, in our analyses, we looked only at frequency of occurrence of individual interventions as in some cases multiple interventions were highlighted in the same report. Third, the selection of eligible reports was performed by only one reviewer and checked by another. However, this pragmatic approach was in agreement with methodological guides on knowledge syntheses due to the low level of complexity regarding our eligibility criteria [100].

## Conclusions

We identified existing effective interventions which could contribute to strengthening the four capacities of the health systems in Africa. Across the 13 elements of health systems, service delivery and health workforce were critical elements, mostly covered by effective interventions for improving the four capacities of health systems. However, service delivery processes and health workforce mobilization can be challenging in underserved settings or where human and health system resources are scarce. In low- and middle-income countries, digital health models and capacity building have been utilized extensively to alleviate these challenges. Thus, to have a significant impact on the functionality of health systems, more attention and investment need to be directed towards scaling of effective interventions covering multiple elements that includes service delivery and health workforce. For example, the focus on health workforce will enhance access to essential services (by ensuring adequate number and skills mix are present where needed), quality of care (by ensuring people-centered care is streamlined), demand for essential services (by providing quality care for services that are requirement by the local population), and resilience (through means of agile, capacitated, and mobile workforce in place). However, effective investment in all 13 elements is a pre-requisite for the ambitious health goals, and it has attempted to provide the first set of effective compendia across the health systems elements and functionality dimensions. Aligned to the Framework of Actions, the set of effective interventions are expected to enhance the functionality of the health systems, which in return builds the foundation required for attainment of universal health coverage, health security, and determinants of health. Finally, further analyses could also deepen understanding of how included interventions differ in their incorporation of evidence about potential for scaling across African countries.

## Supporting information

S1 Appendix Preferred Reporting Items for Systematic reviews and Meta-Analyses extension for Scoping Reviews (PRISMA-ScR) checklist. (DOCX)

S2 Appendix Search strategy for electronic databases. (DOCX)

S3 Appendix Search strategy for grey literature or information. (DOCX)

S4 Appendix List of excluded reports with reason for exclusion. (XLSX)

S5 Appendix Characteristics of each included intervention. (XLSX)
