# Supplementary material for: Investments for effective functionality of health systems towards Universal Health Coverage in Africa: A scoping review
Source: PLOS Glob Public Health. 2022 Sep 23;2(9):e0001076. doi: 10.1371/journal.pgph.0001076 (PMC10021830; doi:10.1371/journal.pgph.0001076)
Supplement: S2 Appendix — (DOCX) [file pgph.0001076.s002.docx]

**S2 Appendix.** Search strategy for electronic databases

**MEDLINE via Ovid (searched on May 3, 2022)**

| **Concept** | **Keyword** | **Number** | **Result** |
| --- | --- | --- | --- |
| **Intervention** | (innovation? OR intervention? OR technolog* OR practice* OR initiative* OR program* OR product? or service* OR strateg* or proces*).ti,ab,sh,kf | 1 | 7,431,457 |
| **Health system functionality** | (("health* system*" OR "care system*") adj3 (function* OR strength* OR reform* OR perform*)).ti,ab,sh,kf | 2 | 7,519 |
| **Low- and middle-income context** | (afghanistan OR albania OR algeria OR american samoa OR angola OR "antigua and barbuda" OR antigua OR barbuda OR argentina OR armenia OR armenian OR aruba OR azerbaijan OR bahrain OR bangladesh OR barbados OR republic of belarus OR belarus OR byelarus OR belorussia OR byelorussian OR belize OR british honduras OR benin OR dahomey OR bhutan OR bolivia OR "bosnia and herzegovina" OR bosnia OR herzegovina OR botswana OR bechuanaland OR brazil OR brasil OR bulgaria OR burkina faso OR burkina fasso OR upper volta OR burundi OR urundi OR cabo verde OR cape verde OR cambodia OR kampuchea OR khmer republic OR cameroon OR cameron OR cameroun OR central african republic OR ubangi shari OR chad OR chile OR china OR colombia OR comoros OR comoro islands OR iles comores OR mayotte OR democratic republic of the congo OR democratic republic congo OR congo OR zaire OR costa rica OR "cote d’ivoire" OR "cote d’ ivoire" OR cote divoire OR cote d ivoire OR ivory coast OR croatia OR cuba OR cyprus OR czech republic OR czechoslovakia OR djibouti OR french somaliland OR dominica OR dominican republic OR ecuador OR egypt OR united arab republic OR el salvador OR equatorial guinea OR spanish guinea OR eritrea OR estonia OR eswatini OR swaziland OR ethiopia OR fiji OR gabon OR gabonese republic OR gambia OR "georgia (republic)" OR georgian OR ghana OR gold coast OR gibraltar OR greece OR grenada OR guam OR guatemala OR guinea OR guinea bissau OR guyana OR british guiana OR haiti OR hispaniola OR honduras OR hungary OR india OR indonesia OR timor OR iran OR iraq OR isle of man OR jamaica OR jordan OR kazakhstan OR kazakh OR kenya OR "democratic people’s republic of korea" OR republic of korea OR north korea OR south korea OR korea OR kosovo OR kyrgyzstan OR kirghizia OR kirgizstan OR kyrgyz republic OR kirghiz OR laos OR lao pdr OR "lao people's democratic republic" OR latvia OR lebanon OR lebanese republic OR lesotho OR basutoland OR liberia OR libya OR libyan arab jamahiriya OR lithuania OR macau OR macao OR republic of north macedonia OR macedonia OR madagascar OR malagasy republic OR malawi OR nyasaland OR malaysia OR malay federation OR malaya federation OR maldives OR indian ocean islands OR indian ocean OR mali OR malta OR micronesia OR federated states of micronesia OR kiribati OR marshall islands OR nauru OR northern mariana islands OR palau OR tuvalu OR mauritania OR mauritius OR mexico OR moldova OR moldovian OR mongolia OR montenegro OR morocco OR ifni OR mozambique OR portuguese east africa OR myanmar OR burma OR namibia OR nepal OR netherlands antilles OR nicaragua OR niger OR nigeria OR oman OR muscat OR pakistan OR panama OR papua new guinea OR new guinea OR paraguay OR peru OR philippines OR philipines OR phillipines OR phillippines OR poland OR "polish people's republic" OR portugal OR portuguese republic OR puerto rico OR romania OR russia OR russian federation OR ussr OR soviet union OR union of soviet socialist republics OR rwanda OR ruanda OR samoa OR pacific islands OR polynesia OR samoan islands OR navigator island OR navigator islands OR "sao tome and principe" OR saudi arabia OR senegal OR serbia OR seychelles OR sierra leone OR slovakia OR slovak republic OR slovenia OR melanesia OR solomon island OR solomon islands OR norfolk island OR norfolk islands OR somalia OR south africa OR south sudan OR sri lanka OR ceylon OR "saint kitts and nevis" OR "st. kitts and nevis" OR saint lucia OR "st. lucia" OR "saint vincent and the grenadines" OR saint vincent OR "st. vincent" OR grenadines OR sudan OR suriname OR surinam OR dutch guiana OR netherlands guiana OR syria OR syrian arab republic OR tajikistan OR tadjikistan OR tadzhikistan OR tadzhik OR tanzania OR tanganyika OR thailand OR siam OR timor leste OR east timor OR togo OR togolese republic OR tonga OR "trinidad and tobago" OR trinidad OR tobago OR tunisia OR turkey OR turkmenistan OR turkmen OR uganda OR ukraine OR uruguay OR uzbekistan OR uzbek OR vanuatu OR new hebrides OR venezuela OR vietnam OR viet nam OR middle east OR west bank OR gaza OR palestine OR yemen OR yugoslavia OR zambia OR zimbabwe OR northern rhodesia OR global south OR africa south of the sahara OR sub-saharan africa OR subsaharan africa OR africa, central OR central africa OR africa, northern OR north africa OR northern africa OR magreb OR maghrib OR sahara OR africa, southern OR southern africa OR africa, eastern OR east africa OR eastern africa OR africa, western OR west africa OR western africa OR west indies OR indian ocean islands OR caribbean OR central america OR latin america OR "south and central america" OR south america OR asia, central OR central asia OR asia, northern OR north asia OR northern asia OR asia, southeastern OR southeastern asia OR south eastern asia OR southeast asia OR south east asia OR asia, western OR western asia OR europe, eastern OR east europe OR eastern europe OR developing country OR developing countries OR developing nation? OR developing population? OR developing world OR less developed countr* OR less developed nation? OR less developed population? OR less developed world OR lesser developed countr* OR lesser developed nation? OR lesser developed population? OR lesser developed world OR under developed countr* OR under developed nation? OR under developed population? OR under developed world OR underdeveloped countr* OR underdeveloped nation? OR underdeveloped population? OR underdeveloped world OR middle income countr* OR middle income nation? OR middle income population? OR low income countr* OR low income nation? OR low income population? OR lower income countr* OR lower income nation? OR lower income population? OR underserved countr* OR underserved nation? OR underserved population? OR underserved world OR under served countr* OR under served nation? OR under served population? OR under served world OR deprived countr* OR deprived nation? OR deprived population? OR deprived world OR poor countr* OR poor nation? OR poor population? OR poor world OR poorer countr* OR poorer nation? OR poorer population? OR poorer world OR developing econom* OR less developed econom* OR lesser developed econom* OR under developed econom* OR underdeveloped econom* OR middle income econom* OR low income econom* OR lower income econom* OR low gdp OR low gnp OR low gross domestic OR low gross national OR lower gdp OR lower gnp OR lower gross domestic OR lower gross national OR lmic OR lmics OR third world OR lami countr* OR transitional countr* OR emerging economies OR emerging nation?).ti,ab,sh,kf | 3 | 2,212,373 |
| **All** | **1 AND 2 AND 3** | **4** | **3,381** |

**PsycINFO via Ovid (searched on May 3, 2022)**

| **Concept** | **Keyword** | **Number** | **Result** |
| --- | --- | --- | --- |
| **Intervention** | (innovation? OR intervention? OR technolog* OR practice* OR initiative* OR program* OR product? or service* OR strateg* or proces*).ti,ab,sh | 1 | 2,162,271 |
| **Health system functionality** | (("health* system*" OR "care system*") adj3 (function* OR strength* OR reform* OR perform*)).ti,ab,sh | 2 | 1,063 |
| **Low- and middle-income context** | (afghanistan OR albania OR algeria OR american samoa OR angola OR "antigua and barbuda" OR antigua OR barbuda OR argentina OR armenia OR armenian OR aruba OR azerbaijan OR bahrain OR bangladesh OR barbados OR republic of belarus OR belarus OR byelarus OR belorussia OR byelorussian OR belize OR british honduras OR benin OR dahomey OR bhutan OR bolivia OR "bosnia and herzegovina" OR bosnia OR herzegovina OR botswana OR bechuanaland OR brazil OR brasil OR bulgaria OR burkina faso OR burkina fasso OR upper volta OR burundi OR urundi OR cabo verde OR cape verde OR cambodia OR kampuchea OR khmer republic OR cameroon OR cameron OR cameroun OR central african republic OR ubangi shari OR chad OR chile OR china OR colombia OR comoros OR comoro islands OR iles comores OR mayotte OR democratic republic of the congo OR democratic republic congo OR congo OR zaire OR costa rica OR "cote d’ivoire" OR "cote d’ ivoire" OR cote divoire OR cote d ivoire OR ivory coast OR croatia OR cuba OR cyprus OR czech republic OR czechoslovakia OR djibouti OR french somaliland OR dominica OR dominican republic OR ecuador OR egypt OR united arab republic OR el salvador OR equatorial guinea OR spanish guinea OR eritrea OR estonia OR eswatini OR swaziland OR ethiopia OR fiji OR gabon OR gabonese republic OR gambia OR "georgia (republic)" OR georgian OR ghana OR gold coast OR gibraltar OR greece OR grenada OR guam OR guatemala OR guinea OR guinea bissau OR guyana OR british guiana OR haiti OR hispaniola OR honduras OR hungary OR india OR indonesia OR timor OR iran OR iraq OR isle of man OR jamaica OR jordan OR kazakhstan OR kazakh OR kenya OR "democratic people’s republic of korea" OR republic of korea OR north korea OR south korea OR korea OR kosovo OR kyrgyzstan OR kirghizia OR kirgizstan OR kyrgyz republic OR kirghiz OR laos OR lao pdr OR "lao people's democratic republic" OR latvia OR lebanon OR lebanese republic OR lesotho OR basutoland OR liberia OR libya OR libyan arab jamahiriya OR lithuania OR macau OR macao OR republic of north macedonia OR macedonia OR madagascar OR malagasy republic OR malawi OR nyasaland OR malaysia OR malay federation OR malaya federation OR maldives OR indian ocean islands OR indian ocean OR mali OR malta OR micronesia OR federated states of micronesia OR kiribati OR marshall islands OR nauru OR northern mariana islands OR palau OR tuvalu OR mauritania OR mauritius OR mexico OR moldova OR moldovian OR mongolia OR montenegro OR morocco OR ifni OR mozambique OR portuguese east africa OR myanmar OR burma OR namibia OR nepal OR netherlands antilles OR nicaragua OR niger OR nigeria OR oman OR muscat OR pakistan OR panama OR papua new guinea OR new guinea OR paraguay OR peru OR philippines OR philipines OR phillipines OR phillippines OR poland OR "polish people's republic" OR portugal OR portuguese republic OR puerto rico OR romania OR russia OR russian federation OR ussr OR soviet union OR union of soviet socialist republics OR rwanda OR ruanda OR samoa OR pacific islands OR polynesia OR samoan islands OR navigator island OR navigator islands OR "sao tome and principe" OR saudi arabia OR senegal OR serbia OR seychelles OR sierra leone OR slovakia OR slovak republic OR slovenia OR melanesia OR solomon island OR solomon islands OR norfolk island OR norfolk islands OR somalia OR south africa OR south sudan OR sri lanka OR ceylon OR "saint kitts and nevis" OR "st. kitts and nevis" OR saint lucia OR "st. lucia" OR "saint vincent and the grenadines" OR saint vincent OR "st. vincent" OR grenadines OR sudan OR suriname OR surinam OR dutch guiana OR netherlands guiana OR syria OR syrian arab republic OR tajikistan OR tadjikistan OR tadzhikistan OR tadzhik OR tanzania OR tanganyika OR thailand OR siam OR timor leste OR east timor OR togo OR togolese republic OR tonga OR "trinidad and tobago" OR trinidad OR tobago OR tunisia OR turkey OR turkmenistan OR turkmen OR uganda OR ukraine OR uruguay OR uzbekistan OR uzbek OR vanuatu OR new hebrides OR venezuela OR vietnam OR viet nam OR middle east OR west bank OR gaza OR palestine OR yemen OR yugoslavia OR zambia OR zimbabwe OR northern rhodesia OR global south OR africa south of the sahara OR sub-saharan africa OR subsaharan africa OR africa, central OR central africa OR africa, northern OR north africa OR northern africa OR magreb OR maghrib OR sahara OR africa, southern OR southern africa OR africa, eastern OR east africa OR eastern africa OR africa, western OR west africa OR western africa OR west indies OR indian ocean islands OR caribbean OR central america OR latin america OR "south and central america" OR south america OR asia, central OR central asia OR asia, northern OR north asia OR northern asia OR asia, southeastern OR southeastern asia OR south eastern asia OR southeast asia OR south east asia OR asia, western OR western asia OR europe, eastern OR east europe OR eastern europe OR developing country OR developing countries OR developing nation? OR developing population? OR developing world OR less developed countr* OR less developed nation? OR less developed population? OR less developed world OR lesser developed countr* OR lesser developed nation? OR lesser developed population? OR lesser developed world OR under developed countr* OR under developed nation? OR under developed population? OR under developed world OR underdeveloped countr* OR underdeveloped nation? OR underdeveloped population? OR underdeveloped world OR middle income countr* OR middle income nation? OR middle income population? OR low income countr* OR low income nation? OR low income population? OR lower income countr* OR lower income nation? OR lower income population? OR underserved countr* OR underserved nation? OR underserved population? OR underserved world OR under served countr* OR under served nation? OR under served population? OR under served world OR deprived countr* OR deprived nation? OR deprived population? OR deprived world OR poor countr* OR poor nation? OR poor population? OR poor world OR poorer countr* OR poorer nation? OR poorer population? OR poorer world OR developing econom* OR less developed econom* OR lesser developed econom* OR under developed econom* OR underdeveloped econom* OR middle income econom* OR low income econom* OR lower income econom* OR low gdp OR low gnp OR low gross domestic OR low gross national OR lower gdp OR lower gnp OR lower gross domestic OR lower gross national OR lmic OR lmics OR third world OR lami countr* OR transitional countr* OR emerging economies OR emerging nation?).ti,ab,sh | 3 | 251,690 |
| **All** | **1 AND 2 AND 3** | **4** | **387** |

**Cochrane Library (searched on May 3, 2022)**

| **Concept** | **Keyword** | **Number** | **Result** |
| --- | --- | --- | --- |
| **Intervention** | (innovation* OR intervention* OR technolog* OR practice* OR initiative* OR program* OR product* OR service* OR strateg* OR proces*):ti,ab,kw | 1 | 760,863 |
| **Health system functionality** | ("health* system*" OR "care system*") NEAR/2 (function* OR strength* OR reform* OR perform*):ti,ab,kw | 2 | 130 |
| **Low- and middle-income context** | (afghanistan OR albania OR algeria OR "american samoa" OR angola OR "antigua and barbuda" OR antigua OR barbuda OR argentina OR armenia OR armenian OR aruba OR azerbaijan OR bahrain OR bangladesh OR barbados OR "republic of belarus" OR belarus OR byelarus OR belorussia OR byelorussian OR belize OR "british honduras" OR benin OR dahomey OR bhutan OR bolivia OR "bosnia and herzegovina" OR bosnia OR herzegovina OR botswana OR bechuanaland OR brazil OR brasil OR bulgaria OR "burkina faso" OR "burkina fasso" OR "upper volta" OR burundi OR urundi OR "cabo verde" OR "cape verde" OR cambodia OR kampuchea OR "khmer republic" OR cameroon OR cameron OR cameroun OR "central african republic" OR "ubangi shari" OR chad OR chile OR china OR colombia OR comoros OR "comoro islands" OR "iles comores" OR mayotte OR "democratic republic of the congo" OR "democratic republic congo" OR congo OR zaire OR "costa rica" OR "cote d’ivoire" OR "cote d’ ivoire" OR "cote divoire" OR "cote d ivoire" OR "ivory coast" OR croatia OR cuba OR cyprus OR "czech republic" OR czechoslovakia OR djibouti OR "french somaliland" OR dominica OR "dominican republic" OR ecuador OR egypt OR "united arab republic" OR "el salvador" OR "equatorial guinea" OR "spanish guinea" OR eritrea OR estonia OR eswatini OR swaziland OR ethiopia OR fiji OR gabon OR "gabonese republic" OR gambia OR "georgia (republic)" OR georgia OR georgian OR ghana OR "gold coast" OR gibraltar OR greece OR grenada OR guam OR guatemala OR guinea OR "guinea bissau" OR guyana OR "british guiana" OR haiti OR hispaniola OR honduras OR hungary OR india OR indonesia OR timor OR iran OR iraq OR "isle of man" OR jamaica OR jordan OR kazakhstan OR kazakh OR kenya OR "democratic people’s republic of korea" OR "republic of korea" OR north korea OR south korea OR korea OR kosovo OR kyrgyzstan OR kirghizia OR kirgizstan OR "kyrgyz republic" OR kirghiz OR laos OR "lao pdr" OR "lao people's democratic republic" OR latvia OR lebanon OR "lebanese republic" OR lesotho OR basutoland OR liberia OR libya OR "libyan arab jamahiriya" OR lithuania OR macau OR macao OR "republic of north macedonia" OR macedonia OR madagascar OR "malagasy republic" OR malawi OR nyasaland OR malaysia OR "malay federation" OR "malaya federation" OR maldives OR "indian ocean islands" OR "indian ocean" OR mali OR malta OR micronesia OR "federated states of micronesia" OR kiribati OR "marshall islands" OR nauru OR "northern mariana islands" OR palau OR tuvalu OR mauritania OR mauritius OR mexico OR moldova OR moldovian OR mongolia OR montenegro OR morocco OR ifni OR mozambique OR "portuguese east africa" OR myanmar OR burma OR namibia OR nepal OR "netherlands antilles" OR nicaragua OR niger OR nigeria OR oman OR muscat OR pakistan OR panama OR "papua new guinea" OR paraguay OR peru OR philippines OR philipines OR phillipines OR phillippines OR poland OR "polish people's republic" OR portugal OR "portuguese republic" OR "puerto rico" OR romania OR russia OR "russian federation" OR ussr OR "soviet union" OR "union of soviet socialist republics" OR rwanda OR ruanda OR samoa OR "pacific islands" OR polynesia OR "samoan islands" OR "navigator island" OR "navigator islands" OR "sao tome and principe" OR "saudi arabia" OR senegal OR serbia OR seychelles OR "sierra leone" OR slovakia OR "slovak republic" OR slovenia OR melanesia OR "solomon island" OR "solomon islands" OR "norfolk island" OR "norfolk islands" OR somalia OR "south africa" OR "south sudan" OR "sri lanka" OR ceylon OR "saint kitts and nevis" OR "st. kitts and nevis" OR "saint lucia" OR "st. lucia" OR "saint vincent and the grenadines" OR "saint vincent" OR "st. vincent" OR grenadines OR sudan OR suriname OR surinam OR "dutch guiana" OR "netherlands guiana" OR syria OR "syrian arab republic" OR tajikistan OR tadjikistan OR tadzhikistan OR tadzhik OR tanzania OR tanganyika OR thailand OR siam OR "timor leste" OR "east timor" OR togo OR "togolese republic" OR tonga OR "trinidad and tobago" OR trinidad OR tobago OR tunisia OR turkey OR turkmenistan OR turkmen OR uganda OR ukraine OR uruguay OR uzbekistan OR uzbek OR vanuatu OR "new hebrides" OR venezuela OR vietnam OR "viet nam" OR "middle east" OR "west bank" OR gaza OR palestine OR yemen OR yugoslavia OR zambia OR zimbabwe OR "northern rhodesia" OR "global south" OR "africa south of the sahara" OR "sub saharan africa" OR "subsaharan africa" OR "africa, central" OR "central africa" OR "africa, northern" OR "north africa" OR "northern africa" OR magreb OR maghrib OR sahara OR "africa, southern" OR "southern africa" OR "africa, eastern" OR "east africa" OR "eastern africa" OR "africa, western" OR "west africa" OR "western africa" OR "west indies" OR "indian ocean islands" OR caribbean OR "central america" OR "latin america" OR "south and central america" OR "south america" OR "asia, central" OR "central asia" OR "asia, northern" OR "north asia" OR "northern asia" OR "asia, southeastern" OR "southeastern asia" OR "south eastern asia" OR "southeast asia" OR "south east asia" OR "asia, western" OR "western asia" OR "europe, eastern" OR "east europe" OR "eastern europe" OR "developing country" OR "developing countries" OR "developing nation" OR "developing nations" OR "developing population" OR "developing populations" OR "developing world" OR "less developed country" OR "less developed countries" OR "less developed nation" OR "less developed nations" OR "less developed population" OR "less developed populations" OR "less developed world" OR "lesser developed country" OR "lesser developed countries" OR "lesser developed nation" OR "lesser developed nations" OR "lesser developed population" OR "lesser developed populations" OR "lesser developed world" OR "under developed country" OR "under developed countries" OR "under developed nation" OR "under developed nations" OR "under developed population" OR "under developed populations" OR "under developed world" OR "underdeveloped country" OR "underdeveloped countries" OR "underdeveloped nation" OR "underdeveloped nations" OR "underdeveloped population" OR "underdeveloped populations" OR "underdeveloped world" OR "middle income country" OR "middle income countries" OR "middle income nation" OR "middle income nations" OR "middle income population" OR "middle income populations" OR "low income country" OR "low income countries" OR "low income nation" OR "low income nations" OR "low income population" OR "low income populations" OR "lower income country" OR "lower income countries" OR "lower income nation" OR "lower income nations" OR "lower income population" OR "lower income populations" OR "underserved country" OR "underserved countries" OR "underserved nation" OR "underserved nations" OR "underserved population" OR "underserved populations" OR "underserved world" OR "under served country" OR "under served countries" OR "under served nation" OR "under served nations" OR "under served population" OR "under served populations" OR "under served world" OR "deprived country" OR "deprived countries" OR "deprived nation" OR "deprived nations" OR "deprived population" OR "deprived populations" OR "deprived world" OR "poor country" OR "poor countries" OR "poor nation" OR "poor nations" OR "poor population" OR "poor populations" OR "poor world" OR "poorer country" OR "poorer countries" OR "poorer nation" OR "poorer nations" OR "poorer population" OR "poorer populations" OR "poorer world" OR "developing economy" OR "developing economies" OR "less developed economy" OR "less developed economies" OR "lesser developed economy" OR "lesser developed economies" OR "under developed economy" OR "under developed economies" OR "underdeveloped economy" OR "underdeveloped economies" OR "middle income economy" OR "middle income economies" OR "low income economy" OR "low income economies" OR "lower income economy" OR "lower income economies" OR "low gdp" OR "low gnp" OR "low gross domestic" OR "low gross national" OR "lower gdp" OR "lower gnp" OR "lower gross domestic" OR "lower gross national" OR lmic OR lmics OR "third world" OR "lami country" OR "lami countries" OR "transitional country" OR "transitional countries" OR "emerging economies" OR "emerging nation" OR "emerging nations")):ti,ab,kw | 3 | 280,813 |
| **All** | **1 AND 2 AND 3** | **5** | **89** |

**CINAHL via EBSCOhost (searched on May 3, 2022)**

| **Concept** | **Keyword** | **Number** | **Result** |
| --- | --- | --- | --- |
| **Intervention** | TI(innovation* OR intervention* OR technolog* OR practice* OR initiative* OR program* OR product* OR service* OR strateg* OR proces*) | 1 | 711,536 |
| **Health system functionality** | TI("health* system*" OR "care system*") N2 (function* OR strength* OR reform* OR perform*) | 2 | 864 |
| **Low- and middle-income context** | TI(afghanistan OR albania OR algeria OR american samoa OR angola OR "antigua and barbuda" OR antigua OR barbuda OR argentina OR armenia OR armenian OR aruba OR azerbaijan OR bahrain OR bangladesh OR barbados OR republic of belarus OR belarus OR byelarus OR belorussia OR byelorussian OR belize OR british honduras OR benin OR dahomey OR bhutan OR bolivia OR "bosnia and herzegovina" OR bosnia OR herzegovina OR botswana OR bechuanaland OR brazil OR brasil OR bulgaria OR burkina faso OR burkina fasso OR upper volta OR burundi OR urundi OR cabo verde OR cape verde OR cambodia OR kampuchea OR khmer republic OR cameroon OR cameron OR cameroun OR central african republic OR ubangi shari OR chad OR chile OR china OR colombia OR comoros OR comoro islands OR iles comores OR mayotte OR democratic republic of the congo OR democratic republic congo OR congo OR zaire OR costa rica OR "cote d’ivoire" OR "cote d’ ivoire" OR cote divoire OR cote d ivoire OR ivory coast OR croatia OR cuba OR cyprus OR czech republic OR czechoslovakia OR djibouti OR french somaliland OR dominica OR dominican republic OR ecuador OR egypt OR united arab republic OR el salvador OR equatorial guinea OR spanish guinea OR eritrea OR estonia OR eswatini OR swaziland OR ethiopia OR fiji OR gabon OR gabonese republic OR gambia OR "georgia (republic)" OR georgian OR ghana OR gold coast OR gibraltar OR greece OR grenada OR guam OR guatemala OR guinea OR guinea bissau OR guyana OR british guiana OR haiti OR hispaniola OR honduras OR hungary OR india OR indonesia OR timor OR iran OR iraq OR isle of man OR jamaica OR jordan OR kazakhstan OR kazakh OR kenya OR "democratic people’s republic of korea" OR republic of korea OR north korea OR south korea OR korea OR kosovo OR kyrgyzstan OR kirghizia OR kirgizstan OR kyrgyz republic OR kirghiz OR laos OR lao pdr OR "lao people's democratic republic" OR latvia OR lebanon OR lebanese republic OR lesotho OR basutoland OR liberia OR libya OR libyan arab jamahiriya OR lithuania OR macau OR macao OR republic of north macedonia OR macedonia OR madagascar OR malagasy republic OR malawi OR nyasaland OR malaysia OR malay federation OR malaya federation OR maldives OR indian ocean islands OR indian ocean OR mali OR malta OR micronesia OR federated states of micronesia OR kiribati OR marshall islands OR nauru OR northern mariana islands OR palau OR tuvalu OR mauritania OR mauritius OR mexico OR moldova OR moldovian OR mongolia OR montenegro OR morocco OR ifni OR mozambique OR portuguese east africa OR myanmar OR burma OR namibia OR nepal OR netherlands antilles OR nicaragua OR niger OR nigeria OR oman OR muscat OR pakistan OR panama OR papua new guinea OR new guinea OR paraguay OR peru OR philippines OR philipines OR phillipines OR phillippines OR poland OR "polish people's republic" OR portugal OR portuguese republic OR puerto rico OR romania OR russia OR russian federation OR ussr OR soviet union OR union of soviet socialist republics OR rwanda OR ruanda OR samoa OR pacific islands OR polynesia OR samoan islands OR navigator island OR navigator islands OR "sao tome and principe" OR saudi arabia OR senegal OR serbia OR seychelles OR sierra leone OR slovakia OR slovak republic OR slovenia OR melanesia OR solomon island OR solomon islands OR norfolk island OR norfolk islands OR somalia OR south africa OR south sudan OR sri lanka OR ceylon OR "saint kitts and nevis" OR "st. kitts and nevis" OR saint lucia OR "st. lucia" OR "saint vincent and the grenadines" OR saint vincent OR "st. vincent" OR grenadines OR sudan OR suriname OR surinam OR dutch guiana OR netherlands guiana OR syria OR syrian arab republic OR tajikistan OR tadjikistan OR tadzhikistan OR tadzhik OR tanzania OR tanganyika OR thailand OR siam OR timor leste OR east timor OR togo OR togolese republic OR tonga OR "trinidad and tobago" OR trinidad OR tobago OR tunisia OR turkey OR turkmenistan OR turkmen OR uganda OR ukraine OR uruguay OR uzbekistan OR uzbek OR vanuatu OR new hebrides OR venezuela OR vietnam OR viet nam OR middle east OR west bank OR gaza OR palestine OR yemen OR yugoslavia OR zambia OR zimbabwe OR northern rhodesia OR global south OR africa south of the sahara OR sub-saharan africa OR subsaharan africa OR africa, central OR central africa OR africa, northern OR north africa OR northern africa OR magreb OR maghrib OR sahara OR africa, southern OR southern africa OR africa, eastern OR east africa OR eastern africa OR africa, western OR west africa OR western africa OR west indies OR indian ocean islands OR caribbean OR central america OR latin america OR "south and central america" OR south america OR asia, central OR central asia OR asia, northern OR north asia OR northern asia OR asia, southeastern OR southeastern asia OR south eastern asia OR southeast asia OR south east asia OR asia, western OR western asia OR europe, eastern OR east europe OR eastern europe OR developing country OR developing countries OR developing nation* OR developing population* OR developing world OR less developed countr* OR less developed nation* OR less developed population* OR less developed world OR lesser developed countr* OR lesser developed nation* OR lesser developed population* OR lesser developed world OR under developed countr* OR under developed nation* OR under developed population* OR under developed world OR underdeveloped countr* OR underdeveloped nation* OR underdeveloped population* OR underdeveloped world OR middle income countr* OR middle income nation* OR middle income population* OR low income countr* OR low income nation* OR low income population* OR lower income countr* OR lower income nation* OR lower income population* OR underserved countr* OR underserved nation* OR underserved population* OR underserved world OR under served countr* OR under served nation* OR under served population* OR under served world OR deprived countr* OR deprived nation* OR deprived population* OR deprived world OR poor countr* OR poor nation* OR poor population* OR poor world OR poorer countr* OR poorer nation* OR poorer population* OR poorer world OR developing econom* OR less developed econom* OR lesser developed econom* OR under developed econom* OR underdeveloped econom* OR middle income econom* OR low income econom* OR lower income econom* OR low gdp OR low gnp OR low gross domestic OR low gross national OR lower gdp OR lower gnp OR lower gross domestic OR lower gross national OR lmic OR lmics OR third world OR lami countr* OR transitional countr* OR emerging economies OR emerging nation*) | 3 | 216,276 |
| **All** | **1 AND 2 AND 3** | **4** | **61** |

**ERIC via Ovid (searched on May 3, 2022)**

| **Concept** | **Keyword** | **Number** | **Result** |
| --- | --- | --- | --- |
| **Intervention** | (innovation? OR intervention? OR technolog* OR practice* OR initiative* OR program* OR product? or service* OR strateg* or proces*).ti,ab,sh | 1 | 1,063,789 |
| **Health system functionality** | (("health* system*" OR "care system*") adj3 (function* OR strength* OR reform* OR perform*)).ti,ab,sh | 2 | 56 |
| **Low- and middle-income context** | (afghanistan OR albania OR algeria OR american samoa OR angola OR "antigua and barbuda" OR antigua OR barbuda OR argentina OR armenia OR armenian OR aruba OR azerbaijan OR bahrain OR bangladesh OR barbados OR republic of belarus OR belarus OR byelarus OR belorussia OR byelorussian OR belize OR british honduras OR benin OR dahomey OR bhutan OR bolivia OR "bosnia and herzegovina" OR bosnia OR herzegovina OR botswana OR bechuanaland OR brazil OR brasil OR bulgaria OR burkina faso OR burkina fasso OR upper volta OR burundi OR urundi OR cabo verde OR cape verde OR cambodia OR kampuchea OR khmer republic OR cameroon OR cameron OR cameroun OR central african republic OR ubangi shari OR chad OR chile OR china OR colombia OR comoros OR comoro islands OR iles comores OR mayotte OR democratic republic of the congo OR democratic republic congo OR congo OR zaire OR costa rica OR "cote d’ivoire" OR "cote d’ ivoire" OR cote divoire OR cote d ivoire OR ivory coast OR croatia OR cuba OR cyprus OR czech republic OR czechoslovakia OR djibouti OR french somaliland OR dominica OR dominican republic OR ecuador OR egypt OR united arab republic OR el salvador OR equatorial guinea OR spanish guinea OR eritrea OR estonia OR eswatini OR swaziland OR ethiopia OR fiji OR gabon OR gabonese republic OR gambia OR "georgia (republic)" OR georgian OR ghana OR gold coast OR gibraltar OR greece OR grenada OR guam OR guatemala OR guinea OR guinea bissau OR guyana OR british guiana OR haiti OR hispaniola OR honduras OR hungary OR india OR indonesia OR timor OR iran OR iraq OR isle of man OR jamaica OR jordan OR kazakhstan OR kazakh OR kenya OR "democratic people’s republic of korea" OR republic of korea OR north korea OR south korea OR korea OR kosovo OR kyrgyzstan OR kirghizia OR kirgizstan OR kyrgyz republic OR kirghiz OR laos OR lao pdr OR "lao people's democratic republic" OR latvia OR lebanon OR lebanese republic OR lesotho OR basutoland OR liberia OR libya OR libyan arab jamahiriya OR lithuania OR macau OR macao OR republic of north macedonia OR macedonia OR madagascar OR malagasy republic OR malawi OR nyasaland OR malaysia OR malay federation OR malaya federation OR maldives OR indian ocean islands OR indian ocean OR mali OR malta OR micronesia OR federated states of micronesia OR kiribati OR marshall islands OR nauru OR northern mariana islands OR palau OR tuvalu OR mauritania OR mauritius OR mexico OR moldova OR moldovian OR mongolia OR montenegro OR morocco OR ifni OR mozambique OR portuguese east africa OR myanmar OR burma OR namibia OR nepal OR netherlands antilles OR nicaragua OR niger OR nigeria OR oman OR muscat OR pakistan OR panama OR papua new guinea OR new guinea OR paraguay OR peru OR philippines OR philipines OR phillipines OR phillippines OR poland OR "polish people's republic" OR portugal OR portuguese republic OR puerto rico OR romania OR russia OR russian federation OR ussr OR soviet union OR union of soviet socialist republics OR rwanda OR ruanda OR samoa OR pacific islands OR polynesia OR samoan islands OR navigator island OR navigator islands OR "sao tome and principe" OR saudi arabia OR senegal OR serbia OR seychelles OR sierra leone OR slovakia OR slovak republic OR slovenia OR melanesia OR solomon island OR solomon islands OR norfolk island OR norfolk islands OR somalia OR south africa OR south sudan OR sri lanka OR ceylon OR "saint kitts and nevis" OR "st. kitts and nevis" OR saint lucia OR "st. lucia" OR "saint vincent and the grenadines" OR saint vincent OR "st. vincent" OR grenadines OR sudan OR suriname OR surinam OR dutch guiana OR netherlands guiana OR syria OR syrian arab republic OR tajikistan OR tadjikistan OR tadzhikistan OR tadzhik OR tanzania OR tanganyika OR thailand OR siam OR timor leste OR east timor OR togo OR togolese republic OR tonga OR "trinidad and tobago" OR trinidad OR tobago OR tunisia OR turkey OR turkmenistan OR turkmen OR uganda OR ukraine OR uruguay OR uzbekistan OR uzbek OR vanuatu OR new hebrides OR venezuela OR vietnam OR viet nam OR middle east OR west bank OR gaza OR palestine OR yemen OR yugoslavia OR zambia OR zimbabwe OR northern rhodesia OR global south OR africa south of the sahara OR sub-saharan africa OR subsaharan africa OR africa, central OR central africa OR africa, northern OR north africa OR northern africa OR magreb OR maghrib OR sahara OR africa, southern OR southern africa OR africa, eastern OR east africa OR eastern africa OR africa, western OR west africa OR western africa OR west indies OR indian ocean islands OR caribbean OR central america OR latin america OR "south and central america" OR south america OR asia, central OR central asia OR asia, northern OR north asia OR northern asia OR asia, southeastern OR southeastern asia OR south eastern asia OR southeast asia OR south east asia OR asia, western OR western asia OR europe, eastern OR east europe OR eastern europe OR developing country OR developing countries OR developing nation? OR developing population? OR developing world OR less developed countr* OR less developed nation? OR less developed population? OR less developed world OR lesser developed countr* OR lesser developed nation? OR lesser developed population? OR lesser developed world OR under developed countr* OR under developed nation? OR under developed population? OR under developed world OR underdeveloped countr* OR underdeveloped nation? OR underdeveloped population? OR underdeveloped world OR middle income countr* OR middle income nation? OR middle income population? OR low income countr* OR low income nation? OR low income population? OR lower income countr* OR lower income nation? OR lower income population? OR underserved countr* OR underserved nation? OR underserved population? OR underserved world OR under served countr* OR under served nation? OR under served population? OR under served world OR deprived countr* OR deprived nation? OR deprived population? OR deprived world OR poor countr* OR poor nation? OR poor population? OR poor world OR poorer countr* OR poorer nation? OR poorer population? OR poorer world OR developing econom* OR less developed econom* OR lesser developed econom* OR under developed econom* OR underdeveloped econom* OR middle income econom* OR low income econom* OR lower income econom* OR low gdp OR low gnp OR low gross domestic OR low gross national OR lower gdp OR lower gnp OR lower gross domestic OR lower gross national OR lmic OR lmics OR third world OR lami countr* OR transitional countr* OR emerging economies OR emerging nation?).ti,ab,sh | 3 | 112,638 |
| **All** | **1 AND 2 AND 3** | **4** | **4** |

**Web of Science (searched on May 9, 2022)**

| **Concept** | **Keyword** | **Number** | **Result** |
| --- | --- | --- | --- |
| **Intervention** | TS=(innovation$ OR intervention$ OR technolog* OR practice$ OR initiative$ OR program* OR product$ OR service$ OR strateg* OR proces*) | 1 | 17,502,494 |
| **Health system functionality** | TS=(("health* system*" OR "care system*") NEAR/2 (function* OR  strength* OR reform* OR perform*)) | 2 | 7,240 |
| **Low- and middle-income context** | TS=(afghanistan OR albania OR algeria OR american samoa OR angola OR "antigua and barbuda" OR antigua OR barbuda OR argentina OR armenia OR armenian OR aruba OR azerbaijan OR bahrain OR bangladesh OR barbados OR republic of belarus OR belarus OR byelarus OR belorussia OR byelorussian OR belize OR british honduras OR benin OR dahomey OR bhutan OR bolivia OR "bosnia and herzegovina" OR bosnia OR herzegovina OR botswana OR bechuanaland OR brazil OR brasil OR bulgaria OR burkina faso OR burkina fasso OR upper volta OR burundi OR urundi OR cabo verde OR cape verde OR cambodia OR kampuchea OR khmer republic OR cameroon OR cameron OR cameroun OR central african republic OR ubangi shari OR chad OR chile OR china OR colombia OR comoros OR comoro islands OR iles comores OR mayotte OR democratic republic of the congo OR democratic republic congo OR congo OR zaire OR costa rica OR "cote d’ivoire" OR "cote d’ ivoire" OR cote divoire OR cote d ivoire OR ivory coast OR croatia OR cuba OR cyprus OR czech republic OR czechoslovakia OR djibouti OR french somaliland OR dominica OR dominican republic OR ecuador OR egypt OR united arab republic OR el salvador OR equatorial guinea OR spanish guinea OR eritrea OR estonia OR eswatini OR swaziland OR ethiopia OR fiji OR gabon OR gabonese republic OR gambia OR "georgia (republic)" OR georgian OR ghana OR gold coast OR gibraltar OR greece OR grenada OR guam OR guatemala OR guinea OR guinea bissau OR guyana OR british guiana OR haiti OR hispaniola OR honduras OR hungary OR india OR indonesia OR timor OR iran OR iraq OR isle of man OR jamaica OR jordan OR kazakhstan OR kazakh OR kenya OR "democratic people’s republic of korea" OR republic of korea OR north korea OR south korea OR korea OR kosovo OR kyrgyzstan OR kirghizia OR kirgizstan OR kyrgyz republic OR kirghiz OR laos OR lao pdr OR "lao people's democratic republic" OR latvia OR lebanon OR lebanese republic OR lesotho OR basutoland OR liberia OR libya OR libyan arab jamahiriya OR lithuania OR macau OR macao OR republic of north macedonia OR macedonia OR madagascar OR malagasy republic OR malawi OR nyasaland OR malaysia OR malay federation OR malaya federation OR maldives OR indian ocean islands OR indian ocean OR mali OR malta OR micronesia OR federated states of micronesia OR kiribati OR marshall islands OR nauru OR northern mariana islands OR palau OR tuvalu OR mauritania OR mauritius OR mexico OR moldova OR moldovian OR mongolia OR montenegro OR morocco OR ifni OR mozambique OR portuguese east africa OR myanmar OR burma OR namibia OR nepal OR netherlands antilles OR nicaragua OR niger OR nigeria OR oman OR muscat OR pakistan OR panama OR papua new guinea OR new guinea OR paraguay OR peru OR philippines OR philipines OR phillipines OR phillippines OR poland OR "polish people's republic" OR portugal OR portuguese republic OR puerto rico OR romania OR russia OR russian federation OR ussr OR soviet union OR union of soviet socialist republics OR rwanda OR ruanda OR samoa OR pacific islands OR polynesia OR samoan islands OR navigator island OR navigator islands OR "sao tome and principe" OR saudi arabia OR senegal OR serbia OR seychelles OR sierra leone OR slovakia OR slovak republic OR slovenia OR melanesia OR solomon island OR solomon islands OR norfolk island OR norfolk islands OR somalia OR south africa OR south sudan OR sri lanka OR ceylon OR "saint kitts and nevis" OR "st. kitts and nevis" OR saint lucia OR "st. lucia" OR "saint vincent and the grenadines" OR saint vincent OR "st. vincent" OR grenadines OR sudan OR suriname OR surinam OR dutch guiana OR netherlands guiana OR syria OR syrian arab republic OR tajikistan OR tadjikistan OR tadzhikistan OR tadzhik OR tanzania OR tanganyika OR thailand OR siam OR timor leste OR east timor OR togo OR togolese republic OR tonga OR "trinidad and tobago" OR trinidad OR tobago OR tunisia OR turkey OR turkmenistan OR turkmen OR uganda OR ukraine OR uruguay OR uzbekistan OR uzbek OR vanuatu OR new hebrides OR venezuela OR vietnam OR viet nam OR middle east OR west bank OR gaza OR palestine OR yemen OR yugoslavia OR zambia OR zimbabwe OR northern rhodesia OR global south OR africa south of the sahara OR sub-saharan africa OR subsaharan africa OR africa, central OR central africa OR africa, northern OR north africa OR northern africa OR magreb OR maghrib OR sahara OR africa, southern OR southern africa OR africa, eastern OR east africa OR eastern africa OR africa, western OR west africa OR western africa OR west indies OR indian ocean islands OR caribbean OR central america OR latin america OR "south and central america" OR south america OR asia, central OR central asia OR asia, northern OR north asia OR northern asia OR asia, southeastern OR southeastern asia OR south eastern asia OR southeast asia OR south east asia OR asia, western OR western asia OR europe, eastern OR east europe OR eastern europe OR developing country OR developing countries OR developing nation* OR developing population* OR developing world OR less developed countr* OR less developed nation* OR less developed population* OR less developed world OR lesser developed countr* OR lesser developed nation* OR lesser developed population* OR lesser developed world OR under developed countr* OR under developed nation* OR under developed population* OR under developed world OR underdeveloped countr* OR underdeveloped nation* OR underdeveloped population* OR underdeveloped world OR middle income countr* OR middle income nation* OR middle income population* OR low income countr* OR low income nation* OR low income population* OR lower income countr* OR lower income nation* OR lower income population* OR underserved countr* OR underserved nation* OR underserved population* OR underserved world OR under served countr* OR under served nation* OR under served population* OR under served world OR deprived countr* OR deprived nation* OR deprived population* OR deprived world OR poor countr* OR poor nation* OR poor population* OR poor world OR poorer countr* OR poorer nation* OR poorer population* OR poorer world OR developing econom* OR less developed econom* OR lesser developed econom* OR under developed econom* OR underdeveloped econom* OR middle income econom* OR low income econom* OR lower income econom* OR low gdp OR low gnp OR low gross domestic OR low gross national OR lower gdp OR lower gnp OR lower gross domestic OR lower gross national OR lmic OR lmics OR third world OR lami countr* OR transitional countr* OR emerging economies OR emerging nation*) | 3 | 5,878,974 |
| **All** | **1 AND 2 AND 3** | **4** | **3,919** |

**Embase via Elsevier (searched on May 9, 2022)**

| **Concept** | **Keyword** | **Number** | **Result** |
| --- | --- | --- | --- |
| **Intervention** | (innovation$ OR intervention$ OR technolog* OR practice$ OR initiative$ OR program* OR product$ OR service$ OR strateg* OR proces*):ti,ab,kw  (innovation* OR intervention* OR technolog* OR practice* OR initiative* OR program* OR product* OR service* OR strateg* OR proces*):ti,ab,kw | 1 | 10,173,014 |
| **Health system functionality** | (("health* system*" OR "care system*") NEAR/3 (function* OR  strength* OR reform* OR perform*)):ti,ab,kw | 2 | 9,581 |
| **Low- and middle-income context** | (afghanistan OR albania OR algeria OR "american samoa" OR angola OR "antigua and barbuda" OR antigua OR barbuda OR argentina OR armenia OR armenian OR aruba OR azerbaijan OR bahrain OR bangladesh OR barbados OR "republic of belarus" OR belarus OR byelarus OR belorussia OR byelorussian OR belize OR "british honduras" OR benin OR dahomey OR bhutan OR bolivia OR "bosnia and herzegovina" OR bosnia OR herzegovina OR botswana OR bechuanaland OR brazil OR brasil OR bulgaria OR "burkina faso" OR "burkina fasso" OR "upper volta" OR burundi OR urundi OR "cabo verde" OR "cape verde" OR cambodia OR kampuchea OR "khmer republic" OR cameroon OR cameron OR cameroun OR "central african republic" OR "ubangi shari" OR chad OR chile OR china OR colombia OR comoros OR "comoro islands" OR "iles comores" OR mayotte OR "democratic republic of the congo" OR "democratic republic congo" OR congo OR zaire OR "costa rica" OR "cote divoire" OR "cote d ivoire" OR "ivory coast" OR croatia OR cuba OR cyprus OR "czech republic" OR czechoslovakia OR djibouti OR "french somaliland" OR dominica OR "dominican republic" OR ecuador OR egypt OR "united arab republic" OR "el salvador" OR "equatorial guinea" OR "spanish guinea" OR eritrea OR estonia OR eswatini OR swaziland OR ethiopia OR fiji OR gabon OR "gabonese republic" OR gambia OR "georgia (republic)" OR georgia OR georgian OR ghana OR "gold coast" OR gibraltar OR greece OR grenada OR guam OR guatemala OR guinea OR "guinea bissau" OR guyana OR "british guiana" OR haiti OR hispaniola OR honduras OR hungary OR india OR indonesia OR timor OR iran OR iraq OR "isle of man" OR jamaica OR jordan OR kazakhstan OR kazakh OR kenya OR "republic of korea" OR north korea OR south korea OR korea OR kosovo OR kyrgyzstan OR kirghizia OR kirgizstan OR "kyrgyz republic" OR kirghiz OR laos OR "lao pdr" OR latvia OR lebanon OR "lebanese republic" OR lesotho OR basutoland OR liberia OR libya OR "libyan arab jamahiriya" OR lithuania OR macau OR macao OR "republic of north macedonia" OR macedonia OR madagascar OR "malagasy republic" OR malawi OR nyasaland OR malaysia OR "malay federation" OR "malaya federation" OR maldives OR "indian ocean islands" OR "indian ocean" OR mali OR malta OR micronesia OR "federated states of micronesia" OR kiribati OR "marshall islands" OR nauru OR "northern mariana islands" OR palau OR tuvalu OR mauritania OR mauritius OR mexico OR moldova OR moldovian OR mongolia OR montenegro OR morocco OR ifni OR mozambique OR "portuguese east africa" OR myanmar OR burma OR namibia OR nepal OR "netherlands antilles" OR nicaragua OR niger OR nigeria OR oman OR muscat OR pakistan OR panama OR "papua new guinea" OR paraguay OR peru OR philippines OR philipines OR phillipines OR phillippines OR poland OR portugal OR "portuguese republic" OR "puerto rico" OR romania OR russia OR "russian federation" OR ussr OR "soviet union" OR "union of soviet socialist republics" OR rwanda OR ruanda OR samoa OR "pacific islands" OR polynesia OR "samoan islands" OR "navigator island" OR "navigator islands" OR "sao tome and principe" OR "saudi arabia" OR senegal OR serbia OR seychelles OR "sierra leone" OR slovakia OR "slovak republic" OR slovenia OR melanesia OR "solomon island" OR "solomon islands" OR "norfolk island" OR "norfolk islands" OR somalia OR "south africa" OR "south sudan" OR "sri lanka" OR ceylon OR "saint kitts and nevis" OR "st. kitts and nevis" OR "saint lucia" OR "st. lucia" OR "saint vincent and the grenadines" OR "saint vincent" OR "st. vincent" OR grenadines OR sudan OR suriname OR surinam OR "dutch guiana" OR "netherlands guiana" OR syria OR "syrian arab republic" OR tajikistan OR tadjikistan OR tadzhikistan OR tadzhik OR tanzania OR tanganyika OR thailand OR siam OR "timor leste" OR "east timor" OR togo OR "togolese republic" OR tonga OR "trinidad and tobago" OR trinidad OR tobago OR tunisia OR turkey OR turkmenistan OR turkmen OR uganda OR ukraine OR uruguay OR uzbekistan OR uzbek OR vanuatu OR "new hebrides" OR venezuela OR vietnam OR "viet nam" OR "middle east" OR "west bank" OR gaza OR palestine OR yemen OR yugoslavia OR zambia OR zimbabwe OR "northern rhodesia" OR "global south" OR "africa south of the sahara" OR "sub saharan africa" OR "subsaharan africa" OR "africa, central" OR "central africa" OR "africa, northern" OR "north africa" OR "northern africa" OR magreb OR maghrib OR sahara OR "africa, southern" OR "southern africa" OR "africa, eastern" OR "east africa" OR "eastern africa" OR "africa, western" OR "west africa" OR "western africa" OR "west indies" OR "indian ocean islands" OR caribbean OR "central america" OR "latin america" OR "south and central america" OR "south america" OR "asia, central" OR "central asia" OR "asia, northern" OR "north asia" OR "northern asia" OR "asia, southeastern" OR "southeastern asia" OR "south eastern asia" OR "southeast asia" OR "south east asia" OR "asia, western" OR "western asia" OR "europe, eastern" OR "east europe" OR "eastern europe" OR "developing country" OR "developing countries" OR "developing nation" OR "developing nations" OR "developing population" OR "developing populations" OR "developing world" OR "less developed country" OR "less developed countries" OR "less developed nation" OR "less developed nations" OR "less developed population" OR "less developed populations" OR "less developed world" OR "lesser developed country" OR "lesser developed countries" OR "lesser developed nation" OR "lesser developed nations" OR "lesser developed population" OR "lesser developed populations" OR "lesser developed world" OR "under developed country" OR "under developed countries" OR "under developed nation" OR "under developed nations" OR "under developed population" OR "under developed populations" OR "under developed world" OR "underdeveloped country" OR "underdeveloped countries" OR "underdeveloped nation" OR "underdeveloped nations" OR "underdeveloped population" OR "underdeveloped populations" OR "underdeveloped world" OR "middle income country" OR "middle income countries" OR "middle income nation" OR "middle income nations" OR "middle income population" OR "middle income populations" OR "low income country" OR "low income countries" OR "low income nation" OR "low income nations" OR "low income population" OR "low income populations" OR "lower income country" OR "lower income countries" OR "lower income nation" OR "lower income nations" OR "lower income population" OR "lower income populations" OR "underserved country" OR "underserved countries" OR "underserved nation" OR "underserved nations" OR "underserved population" OR "underserved populations" OR "underserved world" OR "under served country" OR "under served countries" OR "under served nation" OR "under served nations" OR "under served population" OR "under served populations" OR "under served world" OR "deprived country" OR "deprived countries" OR "deprived nation" OR "deprived nations" OR "deprived population" OR "deprived populations" OR "deprived world" OR "poor country" OR "poor countries" OR "poor nation" OR "poor nations" OR "poor population" OR "poor populations" OR "poor world" OR "poorer country" OR "poorer countries" OR "poorer nation" OR "poorer nations" OR "poorer population" OR "poorer populations" OR "poorer world" OR "developing economy" OR "developing economies" OR "less developed economy" OR "less developed economies" OR "lesser developed economy" OR "lesser developed economies" OR "under developed economy" OR "under developed economies" OR "underdeveloped economy" OR "underdeveloped economies" OR "middle income economy" OR "middle income economies" OR "low income economy" OR "low income economies" OR "lower income economy" OR "lower income economies" OR "low gdp" OR "low gnp" OR "low gross domestic" OR "low gross national" OR "lower gdp" OR "lower gnp" OR "lower gross domestic" OR "lower gross national" OR lmic OR lmics OR "third world" OR "lami country" OR "lami countries" OR "transitional country" OR "transitional countries" OR "emerging economies" OR "emerging nation" OR "emerging nations"):ti,ab,kw | 3 | 1,051,249 |
| **All** | **1 AND 2 AND 3** | **4** | **2,787** |
