# Supplementary material for: Investments for effective functionality of health systems towards Universal Health Coverage in Africa: A scoping review
Source: PLOS Glob Public Health. 2022 Sep 23;2(9):e0001076. doi: 10.1371/journal.pgph.0001076 (PMC10021830; doi:10.1371/journal.pgph.0001076)
Supplement: S3 Appendix — (DOCX) [file pgph.0001076.s003.docx]

**S3 Appendix.** Search strategy for grey literature or information

| **Website** | **Inquiry (i.e., search terms) used** | **Date** | **Results** |
| --- | --- | --- | --- |
| **Google** | "Intervention" AND "health system" AND "Africa" filetype:pdf | November 30, 2021 | 395,000 |
|  | "Intervention" AND "health system functionality" AND "Africa" filetype:pdf | November 30, 2021 | 2,470 |
|  | "Intervention" AND " health system strengthening" AND "Africa" filetype:pdf | December 1, 2021 | 32,300 |
|  | "Intervention" AND " health system" AND "Africa" AND "Health infrastructure" filetype:pdf | December 1, 2021 | 39.000 |
|  | "Intervention OU Initiative" ET "Système Santé" ET "Afrique" filetype:pdf | December 1, 2021 | 2,630,000 |
| **Google Scholar** | "intervention" AND "health system" AND "human resources" AND "effectiveness" | December 1, 2021 | 43400 |
|  | "intervention" AND "health system" AND "human resources" AND "effectiveness" AND "resilience" | December 2, 2021 | 7,190 |
|  | "intervention" AND "health system" AND "governance" AND "financing" AND "effectiveness" AND "resilience" | December 2, 2021 | 4,600 |
|  | "intervention" AND "health system" AND "health products" AND "effectiveness" AND "resilience" | December 2, 2021 | 449 |
|  | "intervention" AND "health system" AND "public health" AND "effectiveness" AND "resilience" | December 2, 2021 | 23,500 |
|  | "intervention" AND "health system" AND "information management" AND "effectiveness" AND "resilience" | December 2, 2021 | 1,840 |
| **WHO Library** | Health interventions "AND" Health system "AND" Human resources "AND" Effectiveness | December 2, 2021 | 36,530 |
|  | "Intervention" AND "Health system" AND " LMIC" | December 3, 2021 | 2073 |
| **Hinari** | evidence-integration- intervention- health systems | November 30, 2021 | 909 |
|  | cost effectiveness- health systems strengthening-maternal and child health-LMIC | November 30, 2021 | 116 |
|  | access - primary health care services- LMIC | November 30, 2021 | 6,749 |
|  | human centered design-complex health systems | November 30, 2021 | 673 |
|  | supervision-incentives-health workers-quality of care | November 30, 2021 | 7 |
|  | impact-laboratory accreditation-patient care | November 30, 2021 | 32 |
|  | ensuring and improving quality in the context of health system strengthening | November 30, 2021 | 21,929 |
|  | impact, primary care, electronic medical record system | November 30, 2021 | 127,071 |
|  | health systems- resilience- Nigeria | November 30, 2021 | 6,653 |
|  | healthcare financing-universal health coverage | December 1, 2021 | 625 |
|  | Health systems-governance | December 6, 2021 | 14,622 |
|  | Health systems-values and norms | December 6, 2021 | 5,355 |
